# Social Innovation For Health Research: Development of the SIFHR Checklist

**DOI:** 10.1371/journal.pmed.1003788

**Published:** 2021-09-13

**Authors:** Eneyi E. Kpokiri, Elizabeth Chen, Jingjing Li, Sarah Payne, Priyanka Shrestha, Kaosar Afsana, Uche Amazigo, Phyllis Awor, Jean-Francois de Lavison, Saqif Khan, Jana Mier-Alpaño, Alberto Ong, Shivani Subhedar, Isabelle Wachmuth, Luis Gabriel Cuervo, Kala M. Mehta, Beatrice Halpaap, Joseph D. Tucker

**Affiliations:** 1 Faculty of Infectious and Tropical Diseases, London School of Hygiene and Tropical Medicine, London, United Kingdom; 2 Department of Health Behavior, Gillings School of Global Public Health, University of North Carolina, Chapel Hill, North Carolina, United States of America; 3 Social Entrepreneurship to Spur Health (SESH), Global Health Center Office, Guangzhou City, Guangdong Province, Guangzhou, China; 4 Department of Medical Anthropology, School of Global Health, University of North Carolina, Chapel Hill, North Carolina, United States of America; 5 International Diagnostics Centre, Department of Clinical Research, Faculty of Infectious and Tropical Diseases, London School of Hygiene and Tropical Medicine, London, United Kingdom; 6 BRAC James P Grant School of Public Health, BRAC University, Dhaka, Bangladesh; 7 Pan-African Community Initiative on Education and Health (PACIEH), Enugu, Nigeria; 8 Department of Community Health and Behavioral Sciences, Makerere University School of Public Health, Kampala, Uganda; 9 Ahimsa Fund, Lyon, France; 10 BRAC Health Programme, BRAC Centre, Dhaka, Bangladesh; 11 Social Innovation in Health Initiative (SIHI) Philippines Hub, Department of Clinical Epidemiology, College of Medicine, University of the Philippines, Philippines; 12 Alliance for Improving Health Outcomes (AIHO), Quezon City, Philippines; 13 Institute for Global Health Sciences, University of California, San Francisco, San Francisco, California, United States of America; 14 Service Delivery and Safety Department, Health Systems and Innovation, World Health Organization, Geneva, Switzerland; 15 Research for Health, Pan American Health Organization, Washington, DC, United States of America; 16 TDR, the Special Programme for Research and Training in Tropical Diseases, cosponsored by UNICEF, UNDP, the World Bank, and WHO, Geneva, Switzerland; 17 Institute of Global Health and Infectious Diseases, University of North Carolina, Chapel Hill, North Carolina, United States of America

## Abstract

**Background:**

Social innovations in health are inclusive solutions to address the healthcare delivery gap that meet the needs of end users through a multi-stakeholder, community-engaged process. While social innovations for health have shown promise in closing the healthcare delivery gap, more research is needed to evaluate, scale up, and sustain social innovation. Research checklists can standardize and improve reporting of research findings, promote transparency, and increase replicability of study results and findings.

**Methods and findings:**

The research checklist was developed through a 3-step community-engaged process, including a global open call for ideas, a scoping review, and a 3-round modified Delphi process. The call for entries solicited checklists and related items and was open between November 27, 2019 and February 1, 2020. In addition to the open call submissions and scoping review findings, a 17-item Social Innovation For Health Research (SIFHR) Checklist was developed based on the Template for Intervention Description and Replication (TIDieR) Checklist. The checklist was then refined during 3 rounds of Delphi surveys conducted between May and June 2020. The resulting checklist will facilitate more complete and transparent reporting, increase end-user engagement, and help assess social innovation projects. A limitation of the open call was requiring internet access, which likely discouraged participation of some subgroups.

**Conclusions:**

The SIFHR Checklist will strengthen the reporting of social innovation for health research studies. More research is needed on social innovation for health.

## Abstract 2

Eneyi Kpokiri and co-workers describe development of a research checklist for reporting studies involving social innovations for health.

## Introduction

Social innovations in health are inclusive solutions to address the healthcare delivery gap that meet the needs of end users through a multi-stakeholder, community-engaged process [1]. Many social innovations have been developed in response to specific community needs. A subset of social innovations have transformed health service delivery in low- and middle-income countries (LMICs). For example, social innovations have expanded private sector pharmacy services to manage childhood illnesses in Uganda [1], improved housing, and addressed environmental risks, leading to reduced infestation rates for Chagas disease in Guatemala [2] and increased gonorrhea and chlamydia testing among sexual minorities in China [3]. While these social innovations have shown promise, research is needed to test, implement, adapt, and scale up innovations and their impact [1]. Social innovation in health may strengthen health systems and help to achieve the Sustainable Development Goals of the United Nations Agenda 2030 [1,4].

Research checklists provide one practical way to formalize and standardize the reporting of research findings. Research reporting standards have greatly developed in the past 2 decades, leading to dedicated clearinghouses and collaborations such as the EQUATOR Network, REWARD Campaign, and explicit advocacy and endorsement of such standards in intergovernmental policies and high-level documents aimed at increasing the value of research and reducing research waste [5–8]. Research checklists can spur multidisciplinary research [9,10], increase transparency [9,11,12], improve reporting completeness[9,11–13], and facilitate easier comparison and replicability of study results and findings [9,13,14]. While some checklists are focused on reporting methods [14] and others focus more on the details in reporting results [13], there are some checklists that report on both methods and results [11]. Overall, these checklists help researchers plan, execute, and report their processes and outcomes. However, to our knowledge, there has been only one research checklist that focuses on similar issues in global health [9]. In addition, meetings led by the Social Innovation in Health Initiative (SIHI), a network of international partners convened by TDR (the Special Programme for Research and Training in Tropical Diseases, cosponsored by UNICEF, UNDP, the World Bank, and WHO), highlight the need for research tools to advance social innovation in healthcare delivery in LMICs [15–18]. The purpose of this manuscript is to describe the development of a research checklist to assess and report social innovation projects as well as highlight the importance of research in social innovation projects.

## Methods

**Fig 1:**
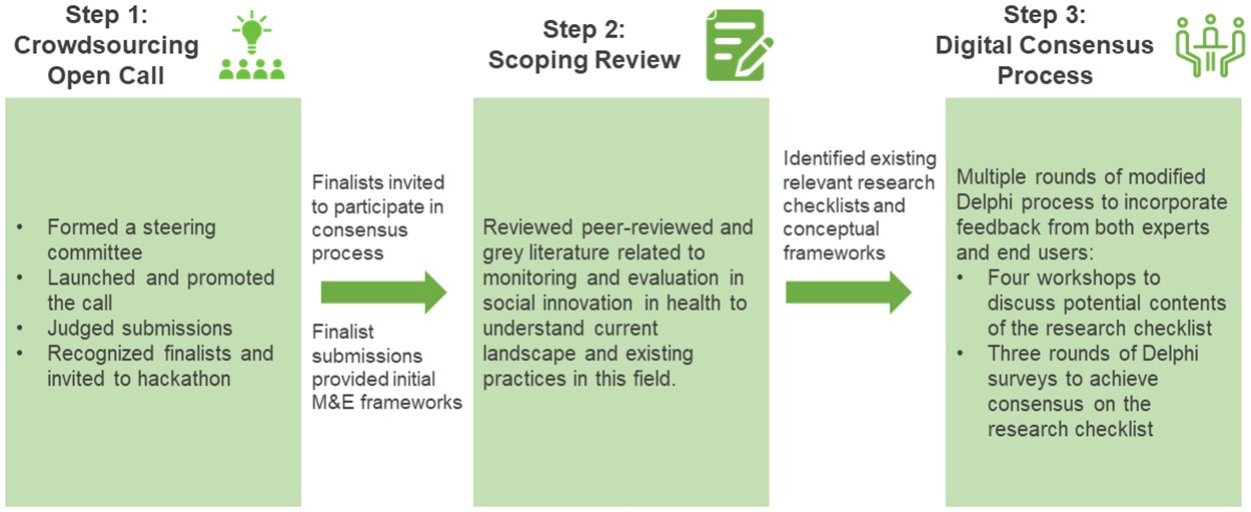
Overview of the process of developing consensus. M&E, monitoring and evaluation.

Our working group used a 3-step process, including an open call for ideas, a scoping review, and a modified Delphi process (Fig 1). This 3-step process resulted in the development of a Social Innovation For Health Research (SIFHR) Checklist as well as a Social Innovation in Health Monitoring and Evaluation Framework [19].

### Open call

Social Entrepreneurship to Spur Health (SESH) is the research hub in China within the TDR SIHI. SESH and SIHI jointly organized a global crowdsourcing open call to solicit creative ideas and tools on the development of a social innovation research checklist, as well as ideas on measuring social innovation in health performance to develop a conceptual framework for measurement and evaluation. Crowdsourcing open calls invite individuals or groups to solve a problem together and then share the solutions with the public [20]. The purpose of the checklist was to develop a list of key components related to social innovation in health research. The measurement ideas were to help project managers and their teams effectively implement their social innovation projects, guide and improve project design, and allow them to more accurately report and measure the impact of their projects.

We formed a steering committee (JT, IW, BH, JF, PA, KM, KA, EK, UA, and SP) to finalize the call for submissions, decide the prize structure, identify judges, and advise on implementation. Steering committee members for this open call included researchers, innovators, policymakers, implementers, and students. This process was similar to other crowdsourcing open calls organized by SESH to understand research mentorship in LMICs [21] and to promote HIV testing and hepatitis testing where online open calls led to in-person consensus building meetings for further action [22,23].

The open call was launched on November 27, 2019 and closed on February 1, 2020. During this time, the open call was distributed within the SIHI network, through social media channels (e.g., Twitter), on SESH’s website, and through other partner and academic networks. The open call solicited monitoring and evaluation frameworks, research checklists, and methods for assessing monitoring and evaluation. Eligibility criteria included written in English, less than 1,000 words, and contained a document or attachment that provided a rationale and explanation for either a monitoring and evaluation framework or a research checklist. Volunteer judges were selected, with a focus on people in LMICs who have experience in social innovation. The focus on strong participation from LMICs was because social innovations are community engaged and locally driven. Too often in global health, high-income country researchers make key decisions that influence the process and outcomes. Our intention of ensuring strong LMIC participation within the steering committee and judging group was to increase the likelihood that this research checklist would be relevant to many people in LMICs. After the open call was closed, each submission was screened independently for eligibility, and eligible entries were reviewed by 5 independent judges. The open call can be found in S1 Text.

Entries were subjectively judged by participants in 3 categories: (1) relevance to inform a standardized framework or research checklist; (2) creativity; and (3) the participant’s experience in the field of social innovation. Scores were assigned between “1” and “10” in each category and then averaged for a final score of the entry. Entries that achieved a mean score of “7” and above were deemed semifinalists. Semifinalists entries were then reviewed once more by the steering committee, and finalists were selected. Finalist submissions were chosen by the steering committee in March 2020 and invited to join a hackathon to finalize the research checklist. Hackathons are a form of crowdsourcing that include an open call for participants, a sprint collaborative event, and follow-up activities [24].

Given the Coronavirus Disease 2019 (COVID-19) pandemic, we transitioned our originally planned in-person workshop to a digital consensus building process following the open call. We asked participants to choose between a day-long videoconference versus shorter sessions over 2 to 3 days and most wanted the latter. We organized an intensive period of collaboration via videoconference calls over the span of several weeks plus 2 separate 2-hour videoconference workshops. We scheduled an additional videoconference focused on introductions and logistics. Further details about the hackathon’s digital consensus building process are described in the section on the modified Delphi process below.

### Scoping review

The steering committee reviewed peer-reviewed literature and gray literature related to social innovation in health to understand the current landscape and existing research and practice efforts in this field. We organized a scoping review based on the framework outlined by Arksey and O’Malley and used the Preferred Reporting Items for Systematic Reviews and Meta-Analyses (PRISMA) Checklist for scoping reviews. The purpose of a scoping review is to examine the depth, breadth, and nature of research on a given topic. This scoping review focused on the monitoring and evaluation related to social innovation in health. We searched PubMed, Cochrane, ClinicalTrials.gov, the EQUATOR Network database, Google Scholar, and Scopus. Included publications examined social innovation in health from the perspective of monitoring and evaluation, with a particular focus on informing a conceptual framework and research checklist. This included conceptual frameworks, research checklists, empirical monitoring studies, evaluation approaches, and similar types of articles. The search strategy included variations of the following terms: social innovation, monitoring, evaluation, conceptual framework, and research checklist. We exported records to EndNote.

Inclusion criteria were conceptual frameworks or research checklists focused on or related to social innovation in health. Studies related to social innovation that mentioned health, but did not focus on health, were also included. We included checklists relevant to the monitoring and evaluation of social innovation projects and research. We excluded records with minimal relevance to social innovation, those only related to programs, systematic, and scoping reviews, and manuscripts not written in English.

JT and EK independently reviewed titles and abstracts for inclusion. Discrepancies were brought to the SIHI working group on monitoring and evaluation. Full-text articles of relevant manuscripts were shared with the entire working group using file sharing software. The initial search was performed on May 5, 2020 and updated on November 30, 2020.

### Checklist development

We followed recommendations from the Guideline International Network [25] and completed a checklist relevant to guideline development (S2 Text) [26].

### Modified Delphi process

The Delphi process is a structured method to develop consensus and is commonly used to develop health guidelines and research checklists [26]. A typical Delphi process has a group of experts iteratively develop a consensus. Given the importance of end users in social innovation, our Delphi process was modified to incorporate feedback from expert (all 3 rounds) and end users (rounds 1 and 2). The expert group consisted of the steering committee and finalists from the crowdsourcing open call. The user group included people with experience and/or interest in social innovation research. Iterative feedback from each of the 3 Delphi surveys (S3 Text) was used to revise the research checklist and the monitoring and evaluation conceptual framework. In the first round, comments suggested the need for more details on the methodology, including examples and key definitions (S4 Text). In round 2, feedback highlighted the need for social innovation impacts, strengths, limitations, and end-user perspectives. The third and final round refined the format of the research checklist. Our team reviewed and analyzed the results of the online surveys between each round of voting.

## Results

### Open call

We received a total of 21 unique submissions from 12 different countries: United States of America (*n* = 5), Bangladesh (*n* = 3), Colombia (*n* = 2), Nigeria (*n* = 2), Philippines (*n* = 2), Cameroon (*n* = 1), Guinea (*n* = 1), Honduras (*n* = 1), India (*n* = 1), Kenya (*n* = 1), Thailand (*n* = 1), and United Kingdom (*n* = 1). Therefore 65% (11 out of 17) of the unique submissions (all those except entries submitted from the USA and the UK) were from LMICs. After the initial screening, 17 out of the 21 submissions were deemed eligible for judging. After the steering committee discussion, 4 finalists were selected: 2 from the USA, 1 from the Philippines, and 1 from Bangladesh.

We noted several themes across entries received, including the following: a strong focus on community and stakeholder engagement; considering implementation as an essential component; and examining financial models and financial sustainability. The eligible entries provided initial frameworks to examine social innovation in health projects at different stages and suggested processes and data that should be reported to enable evaluation of project effectiveness and impact.

### Discussions at workshops

Two 2-hour online workshops were organized on May 19 and May 29 of 2020. The videoconference workshops provided an opportunity to discuss the research checklist based on data from the open call and the findings of the scoping review. Participants included steering committee members and open call finalists. For example, one of the major topics of discussion at our second meeting focused on the topic of financing and how sustainability and revenue generation activities are not consistently reported. The discussion uncovered that some participants felt that financing and sustainability should be explicitly included in the research checklist. We included this item in the draft research checklist and used the modified Delphi process to determine the content of the final version of the checklist.

### Delphi surveys

In between workshops, social innovation experts, end users, and the broader SIHI network were asked to complete online surveys as part of the modified Delphi process. Experts were involved in all 3 Delphi surveys. End users had a separate group to review and were involved in the first 2 Delphi surveys. The broader SIHI network was consulted in the first survey only. The first Delphi survey was completed by 65 out of 96 (68%) invited participants during May 1 to May 5, 2020. Of these, we had 18 males and 47 females from 20 different countries. There were 18 participants from high-income countries and 47 from LMICs across Africa, Asia, and Latin America. More than half of respondents (65%) had previously done research in social innovation in health. Overall responses included structuring the preamble with mission statement and adding important definitions, specifying and clarifying each checklist item, and defining terms used such as health, stakeholders, facilitators versus providers, and open-access resources. Feedback during the first few consensus building videoconference meetings was further incorporated such as including additional items, limitations, and strengths.

The second Delphi survey was conducted from May 29 to June 2. It was completed by 22 out of 45 (49%) invited participants. An end-user meeting was also convened to solicit innovators perspective into the research checklist elements as a separate digital meeting. Based on feedback, we added more specific descriptions of social innovation, ensured consistency across key terms, and provided illustrative examples.

The final Delphi survey was completed by 16 out of 25 (64%) invited participants. Minor adjustments at this stage included fixing grammatical errors and harmonizing definitions. Some themes and specific feedback received from each round are provided in S4 Text.

#### SIFHR Checklist

Our social innovation in health research checklist uses a variety of terms that are defined differently across disciplines. The social innovation research checklist is adapted from the Template for Intervention Description and Replication (TIDieR) Checklist that focuses on better reporting of interventions.[13] Key terms are defined in Table 1, and we use WHO definition of social innovation.

**Table 1:** Terms and definitions for our SIFHR Checklist.

Term	Definition
Community	People living in the same place or sharing common interests
Cocreation	Collaboration between innovators and end users
End users	Those who directly use the social innovation or are impacted (directly or indirectly) by the social innovation in health
Innovators	Those developing the social innovation
Stakeholders	End users, community members, public sector officials, private sector leaders, civil societies, and other local individuals who have an interest in or are impacted (directly or indirectly) by the social innovation in health
Social innovation in health	Inclusive solutions to address healthcare delivery gap and that meet the needs of those who directly benefit from the solution through a multi-stakeholder, community-engaged process (1)
Provider	The person, group, or organization that designed, developed, or implemented the social innovation in health

SIFHR, Social Innovation For Health Research.

At the end of our multistep process, we finalized a research checklist with 17 items (Table 2, S5 Text). Table 2 includes the social innovation in health research checklist, a description of each of the items, and the percentage of Delphi survey respondents who affirmed that each item should be included in our final survey. To determine interrater reliability, we computed a Cohen kappa coefficient (Cohen’s K = 0.7). We have also included a Supporting information file with the checklist in PDF format along with a list of useful resources and additional information about the SIHI research hubs. We gathered this set of resources from steering committee members and finalists during our checklist development process. In addition, we list 3 examples of a completed checklist in Table 3. They describe social innovation research on Chagas disease in Guatemala [2], maternal health in Uganda [23], and sexual health in China [3].

**Table 2:** SIFHR Checklist.

Item	Item no.	Description	Agreement*
Brief name	1	The title or abstract identified of this social innovation in health research study	100%
Problem	2	Describe the current context, background, and problem addressed by the social innovation from the perspective of the end user	95%
Rationale	3	Describe the rationale for the social innovation, including factors that show a change is needed from the perspective of the end user	100%
Social innovation	4	Describe the key components of the social innovation. This could be accompanied by a detailed description, a photograph, or a figure. Describe each of the processes, activities, and elements used in the social innovation, including any enabling or supporting activities	90%
End users	5	Describe the end users of the social innovation in health. Describe how end users are also direct or indirect beneficiaries of the social innovation	95%
Stakeholder involvement*	6	Describe how local stakeholders, including end users, are involved in the design, development, implementation, and evaluation of the social innovation in health. In addition, describe the role of marginalized/vulnerable individuals or groups (e.g., people with disability or others as defined by the innovators) in these processes	100%
Inputs	7	Describe any physical, digital, or informational materials used or distributed during training, delivery, and/or implementation of in the social innovation; provide information on where the materials can be accessed^†^ (e.g., online, appendix, and URL)	95%
Provider	8	For each category of the social innovation provider (e.g., community member, trained layperson, and other individual), describe their expertise, background, role, and any specific training given	90%
Implementation strategy	9	Describe the implementation strategy for the social innovation and whether it is delivered individually, as a group, or partnership. Describe the level of external resources for implementation (e.g., internet access). Describe the frequency and duration of the social innovation delivery	100%
M&E strategy	10	Describe what is measured, how, and when as part of monitoring and evaluation. This includes measurement of health, social, and other impacts	100%
Setting	11	Describe the population and type(s) of location(s) where the social innovation is delivered, including any necessary social, political, cultural, environmental, or other contextual issues. Describe at what level the innovation is implemented (e.g., district, subdistrict, and village). This includes a description of the online setting for online social innovation	90%
Adaptability	12	Consider how the social innovation could be adapted, scaled up, or used in contexts other than the one described, if appropriate	100%
Financing	13	Describe how the social innovation in health has been funded at the design, development, implementation, and evaluation stages. Describe how the social innovation could generate revenue (if applicable) or be institutionalized (if applicable) in order to be sustained in the future	86%
Health impact	14	Describe the health impact of the social innovation over a period of time and the methods to assess health impact. Health is defined broadly here according to WHO definition	100%
Social impact	15	Describe the nonmedical impact of the social innovation over a period of time. This could be impact on the environment, social changes, or other nonmedical impact (e.g., lessons learned, new processes that emerged from the project, new relationships and networks, and application of learned processes to other problems)	100%
Limitations	16	Describe the limitations and potential unintended consequences of the social innovation in health during the design, development, or implementation.	95%
Strengths	17	Describe how the social innovation in health improves on conventional practice	95%

* Denotes percent agreement in the final Delphi survey.

^†^ Open access supplementary material is preferred.

M&E, monitoring and evaluation; SIFHR, Social Innovation For Health Research.

**Table 3:** Examples of social innovations in health described using SIFHR Checklist.

Item	Research checklist item	Castro-Arroyave and colleagues (2020) [2]	Awor and colleagues (2020) [23]	Yang and colleagues (2020) [3]
1	Brief name	Integrated vector control of Chagas disease	ITWA	Pay it forward to increase STI testing among MSM in China
2	Problem	Chagas disease affects about 6 million people, and some 65 million people are at risk of contracting the disease. Chagas disease is a zoonosis that is strongly associated with poverty in rural Latin America. Houses made of adobe or plant material, common in rural Latin America, provide a perfect habitat for triatomine bugs, the vectors of Chagas disease.	Uganda has only one radiologist/sonographer per 1 million people. Combined with lack of advanced imaging technology and low incomes, rural populations greatly lack access to diagnostic imaging services, for example, for timely diagnosis and treatment of pregnancy complications. This can increase the risk of severe illness and death in pregnant women.	WHO recommends that MSM receive gonorrhea and chlamydia testing, but many evidence-based preventative services need to be paid out of pocket, creating financial barriers and health inequity for the poor. In China, dual gonorrhea and chlamydia tests are available in many Chinese hospitals for approximately US$22, yet the testing rate among Chinese MSM are low (12.5% for gonorrhea and 18.1% for chlamydia).
3	Rationale	SIHI hubs can be used for generating new solutions. Partners developed a call to identify social innovation initiatives in health in Central America in 2017 related to CHAGAS. “The knowledge acquired by researchers from University of San Carlos (USAC) in Guatemala about how to improve houses with local material, to avoid the colonization by triatomine bugs that transmit Chagas disease, gave rise to the need to transcend the traditional vision of research and to move toward a perspective that involves the community, promoting their empowerment and participation.”	ITWA is a Ugandan-registered NGO that focuses on incorporating low-cost ultrasound services into remote healthcare facilities where imaging infrastructure is weak where there are no radiologists. By bringing obstetric imaging services closer to rural women, ITWA’s program can help timely identification and treatment of pregnancy complications.	Innovative strategies to expand access to preventive services like gonorrhea and chlamydia testing are needed, especially in LMICs. Public sector responses to subsidize preventive services are limited and altering prices is difficult. Pay-it-forward strategy has the potential to increase trust and community engagement in health services and help reduce the financial barriers to testing.
4	Social innovation	The project was an effective and innovative social approach for the control and prevention of Chagas disease in the municipality of Comapa, Guatemala. The approach consisted in designing a strategy to address predetermined risk factors for the colonization of dwellings by the vectors. The interventions included filling the cracks and crevices in the floors and walls using a combination of locally available materials, raising awareness, and training of leaders and members of the community to adopt the home improvements and contribute to cultural changes such as maintaining animals outside homes to eliminate the risk of colonization of homes by triatomine vectors.	ITWA is a social enterprise and it applies commercial approaches to maximize access to affordable imaging services remote and underserved populations. Their model incorporates the use of ultrasound imaging devices at the point of care, training midwives and nurses (nonradiographers) to conduct ultrasound scans and real time off-site radiology review of the scan by experts (using telemedicine approaches). Together, the use of technology/telemedicine, provision of affordable imaging services, training, task shifting and community participation contribute to much better access to imaging services in rural areas.	The pay-it-forward intervention invites MSM who visits a community HIV testing site to also test for gonorrhea and chlamydia. Individuals are told that the testing fee is 150 yuan (US$22) but they can receive a free gift test, because a previous visitor who cared for them donated toward testing fees. After the test, individuals are asked to donate toward future testing for others on a voluntary basis. Compared to the standard-of-care and also the pay-what-you-want arms, pay it forward significantly increased test uptake.
5	End users	Residents of affected communities near Comapa, Guatemala	Low-income pregnant women from rural communities in Uganda	MSM in China
6	Stakeholder involvement	The eco-health approach (based on environmental, social, and biological risk factor management) described here is intersectoral as well as interdisciplinary. This involved financial backing from a variety of sources, university oversight, collaboration and partnership with the Government, Ministry of Health of Guatemala, international NGOs, and local and regional agencies, and local politician involvement.	All the following stakeholders work together to ensure availability and access to the services: the lower-level government and private health facilities that do not routinely provide imaging services; the district health authorities and health workers/midwives who undertake imaging training and the service provision; the expert radiologists in Uganda and abroad; and the low-income mothers who are not able to pay high costs of ultrasound scan services in the private sector.	Throughout the design, development, implementation, and evaluation of the program, community members are closely involved. First, the pay-it-forward program was developed using crowdsourcing (a practice in which a group solves a problem and shares it with the community) to solicit community input. Program procedures were designed iteratively with community partners (including staff members and volunteers from community-based organizations). Second, the name of program in Chinese (the local language) was crowdsourced from the public using an open contest. Third, participants write handwritten postcards to present to subsequent participants to show a sense of care and community. Finally, several of the community members are coauthors of the published research study.
7	Inputs	“Families received training and materials (volcanic ash and lime from nearby areas) to undertake house improvement. The municipality helped supply the volcanic ash (used also in road construction), and personnel in the Ministry of Health learned the procedure and helped in monitoring.”	ITWA utilizes the Digital Imaging and Communications in Medicine software to compress and share ultrasound images via the internet. In addition to the onsite and offsite experts and staff, there must be a cellphone, laptop, internet connection, and the ultrasound machine for use, at the point of care.	In order to carry out the program, a community-based testing site is needed. Community partners need to have trained staff or volunteers to help individuals understand the testing procedures and collect testing samples. A partner local hospital or laboratory is also needed to carry out the lab tests.
8	Provider	University researcher guided, implemented by community members with local leaders. “Overall, the team at LENAP orchestrated the home improvement strategy in rural areas and conducted the laboratory tests, the Ministry of Health continued spraying and providing treatment, while staff at the health center obtained blood samples that are transported to a laboratory, and continuously monitored patients for symptoms of illness. The Mayor’s office provides the transportation of local materials for house improvements in the villages.”	Nurses and midwives are trained and equipped with skills and knowledge to conduct obstetric ultrasound scans. Through the use of their telemedicine platform, the ultrasound images can be immediately viewed and interpreted by volunteer participating radiologists around Uganda.	Researchers, staff, and volunteers at the community-based HIV testing sites were trained with skills and knowledge to help individuals understand testing procedures and collect testing samples. Lab technicians at a local dermatology hospital laboratory carried out nucleic acid amplification testing.
9	Implementation strategy	By reducing the presence of the vector and the risk of Chagas disease in the intervention areas, the eco-health approach created social value in its most evident form: saving lives from preventable deaths. “Inter-disciplinarity was both an input, a methodological approach and a tangible result of this effort to reduce the presence and incidence of Chagas disease.” “The eco-health approach (based on environmental, social and biological risk factor management) described here is intersectoral as well as interdisciplinary.”	The implementation strategy combines point of care activities (ultrasound imaging, training, task shifting, and telemedicine) with community engagement and pragmatic funding pricing to promote sustainability.	The program was delivered as part of a research study. Participants were randomized in groups of 10, and men who presented with their partners were assigned to the same group. There is a one-third chance to be assigned to the pay-it-forward arm (the other 2 arms were pay as you want and standard of care). If individuals would like to be tested, they would be tested right away on site. The program ran for approximately 1 month.
10	M&E strategy	Through qualitative informant interview. “Polymerase chain reaction (PCR) techniques allowed the researchers to evaluate changes in the bug’s food source after housing improvement, thereby confirming a reduced risk of human-vector contact.” “Infestation rates decreased dramatically… Spatial analysis of the before and after distribution of vectors.”	Data are routinely collected on selected service provision indicators as well as pricing indicators, for better service provision and for sustainability.	This program was carried out as a randomized controlled trial. The process of design, development, implementation, and evaluation was carefully monitored and documented.
11	Setting	The initiative began in 4 villages and was later scaled up to more than 17 villages in 3 different countries with diverse ecosystems and ethnic populations.	The ITWA diagnostic services are provided in remote and underserved districts in Uganda. Starting from 1 district, growth has continued to at least 6 districts.	This takes place in community-based HIV testing centers in major cities in China (Guangzhou and Beijing).
12	Adaptability	“The housing improvement strategy and other components of the intervention in the field were then implemented and evaluated. This test provided visibility to the changes that the intervention generated in the homes and in the daily lives of communities, and provided the bases to replicate, implement and scale up the innovation in neighboring countries including El Salvador, Honduras and Nicaragua.”	Since its inception, the ITWA program has been expanded both in terms of geographic areas and the services they provide. The program was expanded to 6 other districts and a total of 11 health facilities by 2016. Wider scale-up is envisioned over the next 5 years. Ultrasound sonography was extended to include echocardiography in selected areas.	Pay-it-forward strategy has the potential to be adapted to other context other than the current one. The program was designed with several aspects to enhance generalizability to other community-based testing sites: No doctors were involved in implementation, protocols were streamlined into routine services, and messaging was simplified. Whether the current program can be adapted to more resource-constrained settings need to be further explored.
13	Financing	Deployed program through international donors. IDRC of Canada funded the development of the innovation and supported the scale up to El Salvador and Honduras (2011); the JICA funded the transfer of the program to Nicaragua (2014).	Funding is a combination of grants (Phillips, Grand Challenges) as well as minimal client contributions for the service.	The program received funding support from the US National Institutes of Health; the Special Program for Research and Training in Tropical Diseases sponsored by UNICEF, UNDP, World Bank and WHO; the National Key Research and Development Program of China; Doris Duke Charitable Foundation; and the SESH Global.
14	Social impact	Eco-social model. Three processes emerged, giving shape to this experience and contributing toward interdisciplinarity, intersectorality, and community empowerment. These 3 processes generated a multidisciplinary research team of dynamic partners in governmental, NGO agencies, academia, and the community. These processes were not just methodological choices and outcomes of an eco-health approach but will also be crucial to future social innovations in health.	The social impact includes improved maternal and health outcomes that directly impact well-being of families; increased number of women seeking ANC; and increased husband/partner involvement in ANC services. With increased awareness, families and husbands became interested in seeing their unborn child through ultrasonography and preparing for the delivery of the baby.	The program promoted community engagement in health services. In China, MSM still face social stigmatization and may face difficulties visiting the clinic for sexual health testing services. By partnering with community-based organizations, the program was able to not only provide affordable testing resources, but also empower the community partners to provide more health services to their community. The pay-it-forward action could also build collective agency and social cohesion. From a policy perspective, this type of program could also be useful as a temporary measure to generate testing demand and build trust in new services, before the introduction of more comprehensive public-funded programs.
15	Health impact	Infestation rates decreased dramatically inside homes and as long as the walls were kept smooth and without crevices, the triatomine bug was unable to establish itself and reproduce within the households. Spatial analysis of the before and after distribution of vectors [21] substantiated this change. Actual incidence of Chagas was not measured.	ITWA has expanded to 11 rural health facilities in Uganda and has trained over 150 health workers and conducted over 200,000 ultrasound scans since 2010. Data are used to aid healthcare decision-making for the individual pregnant woman as well as at the specific health facility level. ITWA reports that results of obstetric ultrasound scans have contributed to improved management in about 23% of the total pregnancies.	Pay-it-forward strategy increased STI testing. A total of 56% men in the pay-it-forward program agreed to receive the gonorrhea and chlamydia test, compared to 46% in the pay-as-you-want group and 18% in the standard-of-care group.
16	Limitations	First, the period of time for researchers to learn about the initiative and conduct interviews with the communities and other partners was short. Second, the household improvement experience for the control of Chagas disease has been transferred to other countries, but in this case study, only the Guatemala initiative was considered—therefore, these results may not be generalizable to other contexts. Third, the researchers/authors recognize that evaluation of the cost–benefit relationship of the intervention could contribute to the replicability and sustainability of social innovation in health initiatives.	Not listed	First, the program was examined in 2 metropolitan cities in China and making inferences to other settings should be done with caution. Second, this program was evaluated in a research context rather than a practice one. The cost-effectiveness analysis used a short-term time zone and did not calculated the disability-adjusted life years averted or quality-adjusted life years gained.
17	Strengths	Using an intersectoral approach, much more than just health outcomes were achieved.	Through task shifting and development of e-Health/telemedicine ultrasound radiology service, the ITWA program made it possible for rural pregnant women to receive timely, affordable care closer to home. The business model and implementation strategy focus on self-sufficiency and sustainability, which together are necessary for scaling up this innovation.	Compared to the conventional approach, pay-it-forward strategy significantly increased testing uptake and was able to reach more members of key population. The program made gonorrhea and chlamydia testing more affordable and accessible.

ANC, antenatal care; IDRC, International Development Research Centre; ITWA, Imaging the World Africa; JICA, Japanese International Cooperation Agency; LMIC, low- and middle-income country; M&E, monitoring and evaluation; MSM, men who have sex with men; NGO, nongovernmental organization; SESH, Social Entrepreneurship to Spur Health; SIHI, Social Innovation in Health Initiative; STI, sexually transmitted infection.

## Discussion

The SIFHR Checklist will help to democratize research in social innovation in health and enhance the rigor of research on social innovation in health. It is intended for research on social innovation in diverse global settings, especially in LMICs. The research checklist will help to structure research studies, standardize research reporting, and provide guidance for routine monitoring and evaluation related to social innovation in health. Our research checklist extends the literature by focusing on social innovation in health, including iterative feedback from end users at multiple steps, and using inclusive crowdsourcing methods.

Our crowdsourcing open call and digital hackathon provided new methods for inclusive end-user feedback, including end users in LMICs. The process of consensus development is typically driven by experts, and many argue that including other stakeholders is essential [25]. Crowdsourcing open call methods have been used in other health research projects to aggregate wisdom from diverse groups of people [27]. The process involved end users at all stages of the project, including the modified Delphi process that finalized the checklist. Given the recognized importance of end users in health [28], our process for consensus development may be relevant to other guideline development at the national or global level.

Our digital hackathon provided an opportunity to transition an in-person method to a series of online workshops. Most hackathons to date have focused on intense in-person collaboration [24]. Potential benefits of the digital hackathon approach include broader inclusion of individuals who would not have been able to join an in-person event, increased time between events to process information and do additional research, and increased capacity to allow real-time participation from people across multiple time zones.

Our research checklist hackathon process has several limitations. First, the standards on research reporting of social innovation are still emerging. Second, the open call required internet access and was likely easier to access among English speaking academic researchers and those with high socioeconomic status; alternative methods to solicit ideas and contributions (e.g., Unstructured Supplementary Service Data [USSD]) could potentially broaden the reach of future open calls and increase contributions from individuals of lower socioeconomic status. Third, we only accepted submissions in English. However, previous global crowdsourcing open calls suggest that when all 6 official languages of WHO are options for submissions, greater than 90% are in English [29].

This research checklist has implications for research and policy. From a research perspective, this checklist will help people in diverse settings to design, implement, and disseminate social innovation in health research. Further research is needed to understand how to measure social innovation in health. Our research checklist raises questions about optimal methods for designing, implementing, and disseminating social innovation in health research. From a policy perspective, our digital hackathon provides an efficient method for collaborative consensus development that is well suited to the COVID-19 era. This could be relevant to policymakers and health leaders organizing consensus processes.

## Conclusions

This 17-item social innovation in health research checklist expands the social innovation literature and will be iteratively improved. The SIFHR Checklist can lead to better health and social outcomes through more complete and transparent reporting of the development, implementation, and evaluation of social innovations in health. SIFHR can be used before, during, and after cocreating social innovations in health. Use of the research checklist will help to increase end-user and stakeholder engagement, increase the rigor of monitoring and evaluation strategies, consider plans for sustainability, and better determine social and health impacts of social innovation. We hope that researchers, innovators, and partners are able to learn more about the processes and results of social innovation for health research projects from each other and that this will drive improved social and health outcomes.

## Supporting information

S1 Text Social Innovation in Health Monitoring and Evaluation open call. (DOCX)

S2 Text Guideline International Network checklist for guideline development. (DOCX)

S3 Text Delphi survey instrument. (DOCX)

S4 Text Feedback obtained from Delphi surveys. (DOCX)

S5 Text SIFHR Checklist. SIFHR, Social Innovation For Health Research.
(PDF)

## Abbreviations

COVID-19: Coronavirus Disease 2019
LMIC: low- and middle-income country
PRISMA: Preferred Reporting Items for Systematic Reviews and Meta-Analyses
SIFHR: Social Innovation For Health Research
SIHI: Social Innovation in Health Initiative
TIDieR: Template for Intervention Description and Replication
USSD: Unstructured Supplementary Service Data
