# Supplementary material for: Social Innovation For Health Research: Development of the SIFHR Checklist
Source: PLoS Med. 2021 Sep 13;18(9):e1003788. doi: 10.1371/journal.pmed.1003788 (PMC8475987; doi:10.1371/journal.pmed.1003788)
Supplement: S1 Text — (DOCX) [file pmed.1003788.s001.docx]

# **S1 Text: Social Innovation in Health Monitoring & Evaluation open call**

##### How to Measure Social Innovation in Health?

Are you interested in social innovation in health? Social innovation is a form of problem-solving solutions engaging develop an effective, creative and sustainable framework to monitor and evaluate social innovation in health. Hopefully, soliciting innovative ideas for this framework using a global call for submissions will allow for implementers and researchers to utilize a single, specific, standardized framework for social innovation in health initiatives.

##### Purpose

The purpose of this challenge contest is to solicit creative ideas and tools on the development of a social innovation research checklist, as well as a monitoring and evaluation framework concept. The purpose of the checklist will be to develop a list of key components related to social innovation in health research. The monitoring and evaluation framework will be designed to help project managers effectively implement their social innovation projects, guide and improve project design and allow them to more accurately measure the impact of their projects within communities.

##### Who Can Join?

This call is a global call open to anyone with an interest in social innovation projects in health.

##### Guidelines for Submission

Submissions from participants should include certain aspects of an ideal research checklist and/or potential pieces for a monitoring and evaluating framework. Either parts of a research checklist/ evaluation framework or complete tools are welcome.

Guidelines for Research Checklists:

The research checklist should be tailored to social innovation in health and should identify certain steps and/or actions that would guide researchers through the process of analyzing and reporting their findings. Checklists could include any aspects but not limited to different stages of a research project (e.g., ethical approval, study proposal, statement of purpose, research design, risk assessment, instruments or tools etc.), certain ideas or concepts that should be considered when completing this type of research and/or items that may need to be verified or inspected during the research process. The checklist should aim to serve as both a guide and a useful resource for researchers looking specifically at social innovation in health.

Guidelines for Monitoring and Evaluating Frameworks:

communities that prioritize impact and sustainability, while aiming to create positive social changei. Would you like to help improve our understanding of measuring social innovation in health by helping to create standardized tools of assessment? Join our challenge contest!

##### Background

Social innovation is increasingly used to solve important health issues. For example, “Riders for Health” is a global programme selected by the Social Innovation in Health Initiatives (SIHI) to showcase the value of social innovation in health where access to healthcare is improved in rural African communities by providing transportation services that were otherwise unavailableii. However, currently there are numerous forms of innovations in a wide range of domains, but there is no standardized framework to measure the success of social innovation in health, specifically. Being able to measure the impact of social innovation on communities and individuals is just as important as the innovation itself. In order to maximize the positive impact social innovation can have in the healthcare industry and minimize the financial burden of these projects, we must

The monitoring and evaluation framework should look to establish and clearly explain a list of different aspects and/or criteria that would be necessary when measuring or evaluating the impact of a social innovation project. These methods of evaluation should take into account the complexities involved when addressing health issues embedded in larger social contexts and how best to measure the success of projects implemented to address these issues. Submissions could address the following questions: how best to evaluate a social innovation health project? How does one choose the metrics that will be used to evaluate these projects? When reporting research evaluating social innovation in health, what are the essential components? Again, a complete and finalized framework is not expected. Certain pieces or aspects of a framework along with a rationale for why they should be included in this proposed framework are welcome.

##### Format and Requirements for Submissions

1. Submissions in all Six official languages of the WHO will be accepted, but with a preference for English and should be no more than 1,000 words.
2. All submissions should be uploaded as a Word document or PDF file.
3. Use of pictures, tables and charts are welcome, but not compulsory.
4. Checklist items should include a brief note explaining why each particular item is useful for social innovation in health research.
5. A rationale should be given for each aspect of the monitoring and evaluating framework, defending why it would be beneficial to include that particular piece in the framework.
6. If you decide to submit more than one submission, please submit them separately.

##### Judging Submissions

Each submission will be screened independently for eligibility and merit. This will be based on whether or not submissions are in accordance with the format and requirement guidelines stated above. Submissions that are deemed eligible will then be judged based on creativity and their potential to inform and establish a checklist or a framework for social innovation. Judging will also include a second phase, in which the best submissions will go through an in-depth evaluation, including feedback. Judges will include experts in the field of social innovation.

##### Prizes

Finalists will be supported to attend an in-person workshop in May 2020 in Annecy. This consensus building workshop will look to integrate all the best aspects of the different submissions to develop a WHO/TDR/SIHI/SESH guide, where finalists would also be acknowledged.

Finalist submissions will be chosen after being screened and judged by the steering committee. Exceptional submissions will also be announced to the public.

All participants and finalists will also be given an official commendation certificate of recognition, from the WHO/TDR, SIHI and SESH organizing team.

##### TDR

TDR, the Special Program for Research and Training in Tropical Diseases, is a global program of scientific collaboration that helps coordinate, support and influence global efforts to combat infectious diseases of poverty. Established in 1975, TDR is co-sponsored by the United Nations Children’s Fund (UNICEF), the United Nations Development Program (UNDP), the World Bank and the World Health Organization (WHO) and is hosted at WHO. For more information, visit [www.who.int/tdr.](http://www.who.int/tdr) The Social Innovation in Health Initiative (SIHI) utilizes their skills and resources to advance social innovation in health by creating sustainable development, as well as working to improve lives and communities in low- and middle-income countries. SIHI has hubs all over the globe working towards this goal.

##### SIHI

The Social Innovation in Health Initiative (SIHI) is a global network of passionate individuals and institutions combining their skills and resources in support of key activities to promote social innovation in health. The common goal to advance social innovation in achieving Sustainable Development Goals and improving lives of

communities in low- and middle-income countries. This is done through south-south-north collaboration in three areas research, capacity building and advocacy.

##### SESH

SESH, Social Entrepreneurship to Spur Health, is a partnership between universities focused on using crowdsourcing methods to improve health. SESH was founded in 2012 and has organized over 50 crowdsourcing challenge contests. SESH partnered with TDR to organize the Women Leaders in Global Health Challenge Contest in 2018.

##### Timeline

**The deadline for submissions is January 15th, 2020. No entries will be accepted once the deadline has passed.**

##### Submitting Entries

Participants can submit their entries to the following link:

Social Innovation in Health Challenge Contest Survey

All entries must be submitted by the deadline of **January 15th 2020**. If you cannot access the Qualtrics survey, please email your submission to the following address by the deadline: [siframeworkchallenge@gmail.com](mailto:siframeworkchallenge@gmail.com)

##### Questions

If you have any questions or inquiries, please contact the challenge coordinator at: [siframeworkchallenge@gmail.com](mailto:siframeworkchallenge@gmail.com)

##### FAQs:

*What is a challenge competition and open contest?*

Open contests and challenge competitions are ways in which the public is called upon to solve a problem via the solicitation of “ideas, images, or strategies.”

*Who uses challenge competitions and open contests?*

Open contests are promising tools to stimulate innovation and creativity in health and science, and have been utilized by governments, private foundations, and other research institutions across the world.

*What is social innovation?*

Social innovation looks to solve, address, or combat social issues through innovative solutions which include, but are not limited to programs, services, laws, ideas and products. Social innovation focuses on solutions benefiting society, rather than individuals. These innovations aim to solve complex social problems.

*Why should I submit?*

Without globally recognized standards to evaluate social innovation projects in the health field, it will be very difficult to measure success and real impact over time. If you are interested in contributing to sustainable social innovation and helping to find solutions to some of today’s most pressing societal issues, we welcome your creativity and ideas on establishing a groundbreaking set of criteria that will help define new standards in social innovation in health across the globe.

*Is this contest limited to a particular field in social innovation?*

Yes. The aim of this particular contest is to develop a standardized framework for monitoring and evaluating social innovation projects within the health field. However, we are looking for creativity and outside-the-box thinking in these submissions.

*What should be the focus of my submission?*

Your submission should focus on either creating a clear, concise framework that aims to evaluate the social impact and general outcome of social innovation projects and/or a research checklist that aims to generate a

standardized tool for developing a social innovation project. concise and standardized framework in the form of a checklist that is tailored to monitoring and evaluating social innovation projects in the health field.

*What is my target audience?*

Submissions do not have to be tailored to a specific target audience. This ideal framework for evaluating and monitoring is focused on social innovation in health. However, if you choose to create your framework with an intended audience in mind within this field, please indicate this when submitting your entry.

*What should a strong checklist framework look like? Tips for creating one?*

In order to ensure creativity and innovation among the participants and their entries, there are no requirements for creating the checklist or how the checklist should be presented, however it is best to ensure that your entry is developed specifically for social innovation in health. Evaluating a social innovation project will require different criteria than evaluating the success of a technological innovation, for example.

*How can I encourage my peers to join?*

Participation in this challenge contest can be encouraged via in-person and social media mediums. In-person events include lectures, information sessions, and other community events. In addition to email, text messages and listservs, social media such as Facebook and Twitter, can help raise awareness of the contest through engaging posts and other social media announcements, videos and images.

*Do I need to be a qualified medical physician to participate?*

No—anyone with an interest in social innovation is encouraged to participate in this challenge contest and submit their work.

*Can I submit more than one entry?*

Yes—you can submit as many entries as possible. You are more than welcome to submit entries focused just on a monitoring and evaluation framework or just a research checklist, but we welcome entries on both topics, as well as multiple entries on any of the topics.

##### Steering Committee Members

This committee will be composed of social innovation experts and people who have a specific interest for the topic.

##### Confirmed SC for Social Innovation Monitoring and Evaluation Contest

Joseph Tucker, Isabelle Wachsmuth, Beatrice Halpaap, Jean Francois de Lavison, Don Mathanga, Phyllis Awor, Kala Mehta, Kaosar Afsana, Eneyi Kpokiri

Non-Voting Observer: Uche Amazigo Coordinator: Sarah Payne

##### Partner Organizations
