# Supplementary material for: Social Innovation For Health Research: Development of the SIFHR Checklist
Source: PLoS Med. 2021 Sep 13;18(9):e1003788. doi: 10.1371/journal.pmed.1003788 (PMC8475987; doi:10.1371/journal.pmed.1003788)
Supplement: S2 Text — (DOCX) [file pmed.1003788.s002.docx]

# S2 Text: Guideline International Network checklist for guideline development

Version: June 2, 2014

GIN-McMaster Guideline Development Checklist (GDC)

Guideline Development Checklist (GDC)

Click on the titles in the list of topics below or view the bookmarks to go to a specific topic in the checklist. Clicking on the topic title in the checklist will bring you back to the list of topics on this page. Checkboxes are provided for each item to keep track of steps completed or not applicable as well as a section for notes that can be updated while progressing through the guideline development process.

For definitions of the guideline development topics, terms and acronyms appearing throughout the GDC, please download the [glossary.](http://cebgrade.mcmaster.ca/guidecheck.html)

# Guideline Development Topics:

1. [Organization, Budget, Planning and Training](#_bookmark1)
2. [Priority Setting](#_bookmark2)
3. [Guideline Group Membership](#_bookmark3)
4. [Establishing Guideline Group Processes](#_bookmark4)
5. [Identifying Target Audience and Topic Selection](#_bookmark5)
6. [Consumer and Stakeholder Involvement](#_bookmark6)
7. [Conflict of Interest Considerations](#_bookmark7)
8. [(PICO) Question Generation](#_bookmark8)
9. [Considering Importance of Outcomes and Interventions, Values, Preferences and Utilities](#_bookmark9)
10. [Deciding what Evidence to Include and Searching for Evidence](#_bookmark10)
11. [Summarizing Evidence and Considering Additional Information](#_bookmark11)
12. [Judging Quality, Strength or Certainty of a Body of Evidence](#_bookmark12)
13. [Developing Recommendations and Determining their Strength](#_bookmark13)
14. [Wording of Recommendations and of Considerations of Implementation, Feasibility and Equity](#_bookmark14)
15. [Reporting and Peer Review](#_bookmark15)
16. [Dissemination and Implementation](#_bookmark16)
17. [Evaluation and Use](#_bookmark17)
18. [Updating](#_bookmark18)

1

| Completed | Not Applicable | **Guideline Development Steps** | **Source(s)** | **Notes** |
| --- | --- | --- | --- | --- |
| **[1. Organization, Budget, Planning and Training](#_bookmark0)** | | | | |
| ✔ |  | 1. Establish the structure of the guideline development group and determine the roles, tasks, and relationships among the various groups to be involved (e.g. oversight committee/body to direct guideline topic selection and group membership, working group consisting of experts and methodologists to synthesize evidence, a secretariat to provide administrative support, guideline panel to develop recommendations, and stakeholders and consumers for  consultation). (*see Topics* [*3,*](#_bookmark3) [*4*](#_bookmark4) *&* [*6*](#_bookmark6)) | [1-16](#_bookmark19) |  |
| ✔ |  | 2. Perform a thorough assessment of the proposed guideline development project with respect to financial and feasibility issues concerning the guideline development group (e.g. availability of resources to complete the project,  expected commitment from guideline panel and staff, etc.). | [2-8](#_bookmark20)[,11](#_bookmark29)[,15-22](#_bookmark33) |  |
| ✔ |  | 3. Obtain organizational approval to proceed with the guideline project. | [4-7](#_bookmark22)[,10](#_bookmark28)[,11](#_bookmark29)[,13,](#_bookmark31)  [16](#_bookmark34)[,18](#_bookmark36)[,20](#_bookmark38)[,21](#_bookmark39)[,23](#_bookmark41) |  |
| ✔ |  | 4. Prepare a budget for the development of the guideline, outlining the estimated costs for each step (e.g. working group and staff remuneration, outsourcing of certain tasks to outside organizations or groups, travel expenses, publication and dissemination expenses, etc.). | [7](#_bookmark25)[,16](#_bookmark34)[,17](#_bookmark35)[,20](#_bookmark38)[,24](#_bookmark42)[,25](#_bookmark43) |  |
| ✔ |  | 5. Determine whether guideline panel members will be provided any payment or reimbursement for their time or will work as volunteers. | [3](#_bookmark21)[,10](#_bookmark28)[,19](#_bookmark37)[,24](#_bookmark42) |  |
| ✔ |  | 6. Obtain or secure funding for the development of the guideline, with attention to conflict of interest considerations. (*see Topic* [*7*](#_bookmark7)) | [3](#_bookmark21)[,4](#_bookmark22)[,6](#_bookmark24)[,7](#_bookmark25)[,9](#_bookmark27)[,16](#_bookmark34)[,21,](#_bookmark39)  [26](#_bookmark44)[,27](#_bookmark45) |  |

| ✔ |  | 7. Outline and arrange the administrative support that will be required to facilitate the guideline development process (e.g. a secretariat of the working group to organize and obtain declaration of interests, arrange group meetings, etc.). | [2-9](#_bookmark20)[,16](#_bookmark34)[,20](#_bookmark38)[,22](#_bookmark40) |  |
| --- | --- | --- | --- | --- |
| ✔ |  | 8. Plan and prepare for training and support that will be required for those involved in the guideline development process (e.g. conflict of interest related education or training for guideline panel members, teaching sessions for patients to be involved in the guideline group, etc.). (*see Topics* [*4*](#_bookmark4) *&* [*6*](#_bookmark6)) | [1](#_bookmark19)[,2](#_bookmark20)[,5-7](#_bookmark23)[,14](#_bookmark32)[,15,](#_bookmark33)  [23](#_bookmark41)[,24](#_bookmark42)[,28-30](#_bookmark46) | The team provided written and verbal instructions about the process of creating a consensus document. |
| ✔ |  | 9. Set a timeline for the completion of the guideline and target dates for the completion of milestones in the guideline development process. | [2](#_bookmark20)[,4-10](#_bookmark22)[,13](#_bookmark31)[,16-18,](#_bookmark34)  [20-22](#_bookmark38) |  |
|  | ✔ | 10. Determine what, if any, legal considerations are relevant  for the planned guideline (e.g. reimbursement policies for orphan drugs). | [4-7](#_bookmark22)[,20](#_bookmark38)[,21,](#_bookmark39)  [23](#_bookmark41)[,24](#_bookmark42)[,31](#_bookmark49)[,32](#_bookmark50) |  |
|  | ✔ | 11. Prepare a protocol for the entire guideline that can be completed as the project progresses in order to keep the guideline development group on track, including an outline of the overall goals and objectives for the guideline, the timeline, task assignments, steps that will require documentation of decisions, and the proposed methodology for all steps (i.e. those covered in this checklist, for example the methods for forming the guideline group, selection of topics to be covered in guideline, consensus methods, consultation methods,  evidence search and selection methods, etc.). | [3](#_bookmark21)[,5](#_bookmark23)[,7-11](#_bookmark25)[,13-16,](#_bookmark31)  [20](#_bookmark38)[,23](#_bookmark41)[,27](#_bookmark45)[,28,](#_bookmark46)  [33-35](#_bookmark51) |  |
| **[2. Priority Setting](#_bookmark0)** | | | | |
| ✔ |  | 1. Decide on a process for priority setting of guideline topics needed and who will be responsible for directing the process (e.g. priorities set by oversight committee at headquarters of sponsoring organization, priorities referred by government ministries of health or by professional societies). | [4-14](#_bookmark22)[,16](#_bookmark34)[,17,](#_bookmark35)  [19](#_bookmark37)[,20](#_bookmark38)[,25](#_bookmark43)[,36](#_bookmark54)[,37](#_bookmark55) |  |

| ✔ |  | 2. Apply a systematic and transparent process with specific criteria for the proposal of a guideline topic during priority setting (e.g. high prevalence and burden of disease, avoidable mortality and morbidity, high cost, emerging diseases or emerging care options, variation in clinical practice, rapidly changing evidence, etc.). | [3-6](#_bookmark21)[,8-14](#_bookmark26)[,16,](#_bookmark34)  [17](#_bookmark35)[,19](#_bookmark37)[,20](#_bookmark38)[,25,](#_bookmark43)  [36](#_bookmark54)[,37](#_bookmark55) |  |
| --- | --- | --- | --- | --- |
| ✔ |  | 3. Involve appropriate stakeholders in the priority setting process and guideline topic selection (e.g. clinicians, professional societies, policymakers, payers, the public). (*see Topic* [*6*](#_bookmark6)) | [1](#_bookmark19)[,5-8](#_bookmark23)[,10](#_bookmark28)[,12-](#_bookmark30)  [17](#_bookmark30)[,20](#_bookmark38)[,36](#_bookmark54) |  |
| ✔ |  | 4. Consider and decide how different perspectives about the importance and resources required for implementing the guideline recommendations will be considered (e.g. patients, payers, clinicians, public health programs). (*see Topic* [*11*](#_bookmark11)) | [3](#_bookmark21)[,5](#_bookmark23)[,8](#_bookmark26)[,10](#_bookmark28)[,12](#_bookmark30)[,17,](#_bookmark35)  [19-21](#_bookmark37)[,27](#_bookmark45)[,32,](#_bookmark50)  [35](#_bookmark53)[,38](#_bookmark56) |  |
| ✔ |  | 5. Search for any existing up-to-date guidelines covering the proposed topic and assess their credibility (e.g. AGREE II). Determine whether existing guideline(s) can be adapted or if a completely new guideline should be developed. (*see also Topic* [*10*](#_bookmark10)) | [3-14](#_bookmark21)[,16](#_bookmark34)[,17,](#_bookmark35)  [19](#_bookmark37)[,20](#_bookmark38)[,38](#_bookmark56)[,39](#_bookmark57) |  |
| ✔ |  | 6. Discuss the need or opportunity to partner with other organizations that develop guidelines to determine whether a collaborative effort will be sought for the development of the guideline, or any part of the guideline. | [7](#_bookmark25)[,8](#_bookmark26)[,13](#_bookmark31)[,16](#_bookmark34)[,20](#_bookmark38)[,24,](#_bookmark42)  [25](#_bookmark43)[,40](#_bookmark58) |  |
| ✔ |  | 7. Perform a scoping exercise for the proposed guideline topic with respect to implementation issues and barriers to change (e.g. if developed the guideline is likely to improve health outcomes, implementation of healthcare recommendations is feasible, resources are available, etc.). | [3-9](#_bookmark21)[,11](#_bookmark29)[,13-15](#_bookmark31)[,17,](#_bookmark35)  [19-21](#_bookmark37)[,24](#_bookmark42)[,27,](#_bookmark45)  [32](#_bookmark50)[,35](#_bookmark53)[,41](#_bookmark59) |  |
| ✔ |  | 8. Select or provide a consensus method to be used to agree on the priorities set and the guideline topic selected (e.g. voting, Delphi consensus). (*see Topic* [*4*](#_bookmark4)) | [4-6](#_bookmark22)[,13](#_bookmark31)[,17](#_bookmark35)[,20](#_bookmark38)[,36](#_bookmark54) |  |
| ✔ |  | 9. Document the priority setting process and guideline topic selected to ensure transparency. | [4-6](#_bookmark22)[,11](#_bookmark29)[,13,](#_bookmark31)  [17](#_bookmark35)[,20](#_bookmark38)[,36](#_bookmark54) |  |

| **[3. Guideline Group Membership](#_bookmark0)** | | | | |
| --- | --- | --- | --- | --- |
| ✔ |  | 1. Seek multidisciplinary representation for the guideline development group, including members from the target audience, patients and carers, frontline clinicians, content experts, methodology experts, and experts in health economics, to fulfill the roles required (e.g. for the working group, guideline panel). (*see also Topic* [*6*](#_bookmark6)) | [1](#_bookmark19)[,3-7](#_bookmark21)[,9-11,](#_bookmark27)  [13-16](#_bookmark31)[,19-25](#_bookmark37)[,27,](#_bookmark45)  [37](#_bookmark55)[,39](#_bookmark57)[,42](#_bookmark60)[,43](#_bookmark61) |  |
| ✔ |  | 2. Decide on methods for recruitment and enrollment of members for the guideline development group (e.g. widespread advertising of posts, competitive appointment by interview, etc.). | [1](#_bookmark19)[,2](#_bookmark20)[,5](#_bookmark23)[,6](#_bookmark24)[,9-11](#_bookmark27)[,13,](#_bookmark31)  [16](#_bookmark34)[,20](#_bookmark38)[,24](#_bookmark42)[,25](#_bookmark43)[,43](#_bookmark61) |  |
| ✔ |  | 3. Achieve a topic-appropriate balance of expertise and adequate representation for the guideline panel (e.g. experts and primary care physicians who form the target audience, gender and geographical distribution of panel members), which may be iterative if additional members are required as the target audience and topics within the guideline are refined. (*see Topic* [*5*](#_bookmark5)) | [1-8](#_bookmark19)[,10](#_bookmark28)[,13-16](#_bookmark31)[,20,](#_bookmark38)  [24](#_bookmark42)[,25](#_bookmark43)[,27](#_bookmark45)[,37,](#_bookmark55)  [39](#_bookmark57)[,43](#_bookmark61) |  |
| ✔ |  | 4. Consider the optimum group size for the guideline development group, particularly the guideline panel (e.g. too small of a group may lack sufficient experience, content expertise and wide representation, too large of group may lack cohesiveness and effective group interaction). | [1](#_bookmark19)[,2](#_bookmark20)[,4-7](#_bookmark22)[,9](#_bookmark27)[,13-16,](#_bookmark31)  [20](#_bookmark38)[,23](#_bookmark41)[,24](#_bookmark42)[,37](#_bookmark55)[,43](#_bookmark61) |  |
| ✔ |  | 5. Outline roles for the guideline group members and the tasks they will be responsible for (e.g. forming a writing team, group reporter(s) to take meeting minutes and document decisions made, providing methodology consultation, conducting systematic reviews and obtaining other evidence, providing patient perspective, providing  specialist clinician perspective, etc.). | [1](#_bookmark19)[,2](#_bookmark20)[,4-10](#_bookmark22)[,13-16,](#_bookmark31)  [20](#_bookmark38)[,22](#_bookmark40)[,24](#_bookmark42)[,37,](#_bookmark55)  [39](#_bookmark57)[,43](#_bookmark61) |  |
| ✔ |  | 6. Select group leader(s), or chair(s), experienced in group facilitation, maintaining constructive dynamics, identifying and resolving conflicts, remaining neutral and objective, and having methodological expertise and content expertise. | [1](#_bookmark19)[,2](#_bookmark20)[,4-9](#_bookmark22)[,13-16](#_bookmark31)[,20,](#_bookmark38)  [23-25](#_bookmark41)[,37](#_bookmark55)[,39](#_bookmark57)[,43](#_bookmark61) |  |

| ✔ |  | 7. Document the guideline group member selection process and roles to ensure transparency. | [1](#_bookmark19)[,2](#_bookmark20)[,5-8](#_bookmark23)[,13](#_bookmark31)[,16,](#_bookmark34)  [20](#_bookmark38)[,21](#_bookmark39)[,23](#_bookmark41)[,27](#_bookmark45)[,43](#_bookmark61) |  |
| --- | --- | --- | --- | --- |
| **[4. Establishing Guideline Group Processes](#_bookmark0)** | | | | |
| ✔ |  | 1. Establish how and how often communication with guideline panel members and other groups will take place, who will be responsible for making the arrangements, and consider when to deviate from this approach. | [1](#_bookmark19)[,2](#_bookmark20)[,4-6](#_bookmark22)[,8](#_bookmark26)[,9,](#_bookmark27)  [13](#_bookmark31)[,15](#_bookmark33)[,16](#_bookmark34)[,20,](#_bookmark38)  [24](#_bookmark42)[,43](#_bookmark61) |  |
| ✔ |  | 2. Set expectations and awareness of the group process through an introduction, training, and support for the guideline development group members (e.g. setting ideal conditions for group discussion and decision-making). | [1](#_bookmark19)[,2](#_bookmark20)[,4](#_bookmark22)[,6-9](#_bookmark24)[,14,](#_bookmark32)  [16](#_bookmark34)[,19](#_bookmark37)[,23](#_bookmark41)[,24,](#_bookmark42)  [37](#_bookmark55)[,44](#_bookmark62) |  |
| ✔ |  | 3. As part of the training for the guideline development group, ensure that group members understand what the process and proposed methods will be and that they need to be adhered to (e.g. consensus methods that may be used, anonymous or non-anonymous voting, assessment of evidence, group discussion and contributing ideas). | [1](#_bookmark19)[,2](#_bookmark20)[,4](#_bookmark22)[,6-9](#_bookmark24)[,14](#_bookmark32)[,15,](#_bookmark33)  [19](#_bookmark37)[,24](#_bookmark42)[,33](#_bookmark51)[,44](#_bookmark62) |  |
| ✔ |  | 4. Aim to set optimal conditions for group members to be provided equal opportunities to contribute and for their ideas and arguments to be given appropriate consideration (e.g. during group discussion, decision-making, and when formulating recommendations). | [1](#_bookmark19)[,2](#_bookmark20)[,6-9](#_bookmark24)[,13](#_bookmark31)[,16,](#_bookmark34)  [19](#_bookmark37)[,23](#_bookmark41)[,24](#_bookmark42)[,33,](#_bookmark51)  [37](#_bookmark55)[,44](#_bookmark62) |  |
| ✔ |  | 5. Establish methods for dealing with conflict or disputes  among group members and dysfunction in the group process. | [2](#_bookmark20)[,7-9](#_bookmark25)[,13](#_bookmark31)[,15](#_bookmark33)[,16,](#_bookmark34)  [19](#_bookmark37)[,33](#_bookmark51)[,44](#_bookmark62) |  |
| ✔ |  | 6. Provide opportunities for discussion and feedback about the group process throughout the guideline development project. | E*, [6](#_bookmark24) |  |
| ✔ |  | 7. Establish a method for structured and timely distribution and archiving of documents used and produced in the guideline development. | [2](#_bookmark20)[,5](#_bookmark23)[,8](#_bookmark26)[,9](#_bookmark27)[,14-16](#_bookmark32) |  |

| ✔ |  | 8. Set a quorum for meetings (e.g. 75% of group must be present to formulate guideline recommendations), but expect that all group members attend all meetings as far as possible. | [4](#_bookmark22)[,5](#_bookmark23)[,8](#_bookmark26)[,13](#_bookmark31)[,20](#_bookmark38) |  |
| --- | --- | --- | --- | --- |
| ✔ |  | 9. Set or plan meeting times and locations (virtual or in- person) in advance and prepare a scope and specific agenda for each meeting. | [1](#_bookmark19)[,2](#_bookmark20)[,4](#_bookmark22)[,5](#_bookmark23)[,7](#_bookmark25)[,9,](#_bookmark27)  [13-16](#_bookmark31)[,19](#_bookmark37)[,20](#_bookmark38)[,43](#_bookmark61) |  |
| ✔ |  | 10. Keep a record of all meetings with minutes and determine whether or not to make them publically or internally available (e.g. who attended, what was the agenda, what  decisions were made, what next steps will be). | [2](#_bookmark20)[,4](#_bookmark22)[,5](#_bookmark23)[,8](#_bookmark26)[,15](#_bookmark33)[,43](#_bookmark61) |  |
| **[5. Identifying Target Audience and Topic Selection](#_bookmark0)** | | | | |
| ✔ |  | 1. Identify, define and/or review the primary audience (e.g. primary care physicians, health program managers) and secondary audience(s) (e.g. hospital administrators) for  the guideline and determine how many audiences can be addressed with the guideline. | [4](#_bookmark22)[,5](#_bookmark23)[,7](#_bookmark25)[,8](#_bookmark26)[,11](#_bookmark29)[,14-16,](#_bookmark32)  [19](#_bookmark37)[,20](#_bookmark38)[,23](#_bookmark41)[,27](#_bookmark45)[,35,](#_bookmark53)  [37](#_bookmark55)[,39](#_bookmark57)[,42](#_bookmark60)[,45](#_bookmark63) |  |
| ✔ |  | 2. Consult appropriate stakeholders about the target audience(s) identified to ensure they are applicable for the guideline topic and no relevant audience is missed. (s*ee*  *Topic* [*6*](#_bookmark6)) | [4](#_bookmark22)[,14-16](#_bookmark32)[,42](#_bookmark60) |  |
| ✔ |  | 3. Establish a method and criteria to generate and prioritize a candidate list of topics to be addressed within the guideline (e.g. where evidence is most confusing or controversial, where there is currently uncertainty or  inconsistency in practice, questions about screening, diagnosis, and treatment, etc.). | [3-10](#_bookmark21)[,12-16](#_bookmark30)[,19,](#_bookmark37)  [20](#_bookmark38)[,24](#_bookmark42)[,36](#_bookmark54)[,39,](#_bookmark57)  [45](#_bookmark63)[,46](#_bookmark64) |  |
| ✔ |  | 4. Consult appropriate stakeholders to ensure all relevant  topics for the guideline have been identified and will meet the needs of the target audience(s). (s*ee Topic* [*6*](#_bookmark6)) | [4-6](#_bookmark22)[,12-16](#_bookmark30)[,20](#_bookmark38)[,24,](#_bookmark42)  [36](#_bookmark54)[,47](#_bookmark65) |  |
| ✔ |  | 5. Select or provide a consensus development method to be used by the group in agreeing on the final topics selected to be addressed within the guideline (e.g. Delphi method, nominal group technique). | [5](#_bookmark23)[,9](#_bookmark27)[,16](#_bookmark34)[,20](#_bookmark38)[,36](#_bookmark54) |  |

| ✔ |  | 6. Document the processes of identifying the target audience(s) and selection of topics for the guideline to ensure transparency. | [13-16](#_bookmark31)[,20](#_bookmark38)[,27,](#_bookmark45)  [35](#_bookmark53)[,36](#_bookmark54)[,42](#_bookmark60)[,45](#_bookmark63) |  |
| --- | --- | --- | --- | --- |
| **[6. Consumer and Stakeholder Involvement](#_bookmark0)** | | | | |
| ✔ |  | 1. Identify the appropriate stakeholders to involve and consult with in the development of the guideline to incorporate views of all those who might be affected by the guideline (e.g. professional groups, health managers, policy makers, industry representatives). | [1](#_bookmark19)[,3](#_bookmark21)[,6](#_bookmark24)[,9](#_bookmark27)[,11](#_bookmark29)[,13-16,](#_bookmark31)  [19-21](#_bookmark37)[,23](#_bookmark41)[,24,](#_bookmark42)  [27](#_bookmark45)[,29](#_bookmark47)[,30](#_bookmark48)[,32](#_bookmark50)[,39](#_bookmark57) |  |
| ✔ |  | 2. Identify the appropriate consumers to involve and consult with in the development of the guideline (e.g. individual patients, carers who provide non-reimbursed care and support to patients, members of the public as potential patients and as funders of healthcare through taxation, community organizations that represent the interests of patients, and advocates representing the interests of patients and carers). | [1](#_bookmark19)[,6](#_bookmark24)[,7](#_bookmark25)[,9](#_bookmark27)[,11](#_bookmark29)[,14-16,](#_bookmark32)  [21](#_bookmark39)[,23](#_bookmark41)[,24](#_bookmark42)[,27](#_bookmark45)[,29,](#_bookmark47)  [30](#_bookmark48)[,37](#_bookmark55)[,39](#_bookmark57)[,47](#_bookmark65) |  |
| ✔ |  | 3. Establish methods for consumer and stakeholder involvement and maintain a registry of stakeholders for the guideline (e.g. enrollment of consumer and stakeholder members to participate directly on the guideline panel, announce call for separate consumer and stakeholder meeting(s) or workshop(s), electronic distribution of documents and feedback, open period for review of documents and feedback). | [1](#_bookmark19)[,5](#_bookmark23)[,6](#_bookmark24)[,9](#_bookmark27)[,11](#_bookmark29)[,13-16,](#_bookmark31)  [19-21](#_bookmark37)[,23](#_bookmark41)[,24,](#_bookmark42)  [29](#_bookmark47)[,30](#_bookmark48)[,32](#_bookmark50)[,37,](#_bookmark55)  [39](#_bookmark57)[,43](#_bookmark61)[,47](#_bookmark65) |  |
| ✔ |  | 4. Provide information (e.g. training and introduction sessions) for consumers and stakeholders involved directly on the guideline panel to clarify roles and maximize contributions (e.g. evaluating evidence  objectively, avoiding recommendations based on self- interests). | [1](#_bookmark19)[,6](#_bookmark24)[,15](#_bookmark33)[,23](#_bookmark41)[,24](#_bookmark42)[,29,](#_bookmark47)  [32](#_bookmark50)[,37](#_bookmark55)[,47](#_bookmark65) |  |

| ✔ |  | 5. Determine the roles, tasks and timing for consultation with consumers and stakeholders not directly participating on the guideline panel (e.g. at specific milestones during the guideline development process including opportunities to comment on priority setting, topics for the guideline, identifying target audience, identifying patient-important outcomes, identifying additional evidence, point to consequences that the panel has not considered, review the final guideline draft, etc.). | [1](#_bookmark19)[,6](#_bookmark24)[,11](#_bookmark29)[,14-16](#_bookmark32)[,19,](#_bookmark37)  [21](#_bookmark39)[,24](#_bookmark42)[,29](#_bookmark47)[,32](#_bookmark50)[,37,](#_bookmark55)  [39](#_bookmark57)[,48](#_bookmark66) |  |
| --- | --- | --- | --- | --- |
| ✔ |  | 6. Develop or adopt standard templates for consumer and stakeholder input and comments during consultation, with clear instructions or training modules to ensure effective input. | [5](#_bookmark23)[,6](#_bookmark24)[,13](#_bookmark31)[,15](#_bookmark33)[,32](#_bookmark50) |  |
| ✔ |  | 7. Offer adequate time for consumer and stakeholder feedback and consultation. | [13](#_bookmark31)[,15](#_bookmark33)[,29](#_bookmark47)[,32](#_bookmark50) |  |
| ✔ |  | 8. Set a policy and process for handling consumer and stakeholder feedback and dealing with different perspectives (e.g. ensure that diverse perspectives are taken into account in making decisions, provide transparent rationale for judgements made, provide an appeal process for stakeholders, publish consultation comments and the guideline development panel’s  responses). | [5-7](#_bookmark23)[,13](#_bookmark31)[,29](#_bookmark47)[,32](#_bookmark50)[,48](#_bookmark66) |  |
| ✔ |  | 9. Document the enrollment and selection of consumers and stakeholders for the guideline panel and the involvement and consultation with all other consumers and stakeholders to ensure explicit and transparent methods. | [1](#_bookmark19)[,5](#_bookmark23)[,13](#_bookmark31)[,15](#_bookmark33)[,16](#_bookmark34)[,21,](#_bookmark39)  [27](#_bookmark45)[,29](#_bookmark47)[,32](#_bookmark50) |  |
| **[7. Conflict of Interest (COI) Considerations](#_bookmark0)** | | | | |
| ✔ |  | 1. Set a policy for declaration of interests (DOI) of individual participants at admission to the project, including potential guideline panel members prior to their involvement (e.g. what interests should be disclosed, financial, intellectual, academic/clinical, competitive interests of the professional  society). | [4-7](#_bookmark22)[,9](#_bookmark27)[,11](#_bookmark29)[,13-16,](#_bookmark31)  [18](#_bookmark36)[,20](#_bookmark38)[,21](#_bookmark39)[,23-26,](#_bookmark41)  [37](#_bookmark55)[,39](#_bookmark57)[,43](#_bookmark61)[,45,](#_bookmark63)  [49](#_bookmark67)[,50](#_bookmark68) |  |

| ✔ |  | 2. Set a policy for determination of conflicts of interest (COI) and an approach for collecting and updating COI declarations (e.g. how and what level of financial interest should be disclosed, how long a period of time should be covered by the disclosure, who will judge what constitutes a conflict). | [4-7](#_bookmark22)[,9](#_bookmark27)[,11](#_bookmark29)[,13-16,](#_bookmark31)  [18](#_bookmark36)[,20](#_bookmark38)[,21](#_bookmark39)[,23-26,](#_bookmark41)  [37](#_bookmark55)[,43](#_bookmark61)[,45](#_bookmark63)[,49](#_bookmark67)[,50](#_bookmark68) |  |
| --- | --- | --- | --- | --- |
| ✔ |  | 3. Provide clear instructions and training to the potential guideline group members on how to complete the COI disclosure, including a list of the members who must declare COI and the types of interests to declare including examples. | [4-7](#_bookmark22)[,9](#_bookmark27)[,13-16](#_bookmark31)[,18,](#_bookmark36)  [20](#_bookmark38)[,24](#_bookmark42)[,26](#_bookmark44)[,37,](#_bookmark55)  [45](#_bookmark63)[,49](#_bookmark67)[,50](#_bookmark68) |  |
| ✔ |  | 4. Set a policy for management of COI (e.g. individuals with COI not categorically excluded from guideline development but excused from voting on specific recommendations related to the area of conflict, chair should have no COI, evidence summaries prepared by un-conflicted methodologists,). | [4-7](#_bookmark22)[,9](#_bookmark27)[,11](#_bookmark29)[,13-16,](#_bookmark31)  [18](#_bookmark36)[,20](#_bookmark38)[,21](#_bookmark39)[,23-26,](#_bookmark41)  [37](#_bookmark55)[,43](#_bookmark61)[,45](#_bookmark63)[,49](#_bookmark67)[,50](#_bookmark68) |  |
| ✔ |  | 5. Set a policy to manage COI with respect to funding of the guideline development activities (e.g. advocate for public funding, no commercial sponsorship, commercial sponsorship from entities unrelated to topic of guideline, commercial support for non-direct activities such as translation, no single-source sponsor). | [7](#_bookmark25)[,16](#_bookmark34)[,21](#_bookmark39)[,26](#_bookmark44)[,27,](#_bookmark45)  [30](#_bookmark48)[,49](#_bookmark67) |  |
| ✔ |  | 6. Disclose and publish the funding source and describe the role of the sponsors and support provided for the  development of the guideline. | [3](#_bookmark21)[,4](#_bookmark22)[,7](#_bookmark25)[,9](#_bookmark27)[,16](#_bookmark34)[,21](#_bookmark39)[,23,](#_bookmark41)  [24](#_bookmark42)[,26](#_bookmark44)[,27](#_bookmark45)[,30](#_bookmark48)[,35,](#_bookmark53)  [39](#_bookmark57)[,51](#_bookmark69) |  |
| ✔ |  | 7. Explicitly disclose, publish and describe conflicts of interest of the guideline group members, particularly where the conflicts bear on specific recommendations. | [3-7](#_bookmark21)[,9](#_bookmark27)[,11](#_bookmark29)[,14-16,](#_bookmark32)  [20](#_bookmark38)[,21](#_bookmark39)[,23](#_bookmark41)[,24](#_bookmark42)[,26,](#_bookmark44)  [27](#_bookmark45)[,35](#_bookmark53)[,37](#_bookmark55)[,43](#_bookmark61)[,50,](#_bookmark68)  [51](#_bookmark69) |  |
| **[8. (PICO) Question Generation](#_bookmark0)** | | | | |
| ✔ |  | 1. Establish methods for generating the questions for the guideline, prioritizing questions, and selecting and ranking outcomes. | [3-7](#_bookmark21)[,9](#_bookmark27)[,11-16](#_bookmark29)[,19,](#_bookmark37)  [20](#_bookmark38)[,23](#_bookmark41)[,24](#_bookmark42)[,30](#_bookmark48)[,34,](#_bookmark52)  [39](#_bookmark57)[,43](#_bookmark61)[,45](#_bookmark63)[,46](#_bookmark64)[,52](#_bookmark70) |  |

|  | ✔ | 2. Generate and document the key questions (e.g. clinical, health, policy, cost-effectiveness) to be answered in the guideline using a standard format (e.g. PICO) and determine the criteria by which the questions generated will be prioritized if it is not feasible to answer all questions (e.g. survey guideline panel members, survey stakeholders). | [3-7](#_bookmark21)[,9-16](#_bookmark27)[,19](#_bookmark37)[,20,](#_bookmark38)  [23](#_bookmark41)[,24](#_bookmark42)[,27](#_bookmark45)[,30](#_bookmark48)[,34,](#_bookmark52)  [39](#_bookmark57)[,43](#_bookmark61)[,45](#_bookmark63)[,46](#_bookmark64)[,52](#_bookmark70) |  |
| --- | --- | --- | --- | --- |
|  | ✔ | 3. Explicitly describe the population to whom the guideline is meant to apply. Take into consideration specific characteristics of the population, such as prevalence of multiple comorbidities in the population, geographical setting, and equity issues (e.g. plausible reasons for anticipating differential relative effects across disadvantaged and advantaged populations). | [4-9](#_bookmark22)[,11](#_bookmark29)[,12](#_bookmark30)[,14-16,](#_bookmark32)  [19](#_bookmark37)[,20](#_bookmark38)[,23](#_bookmark41)[,24](#_bookmark42)[,27,](#_bookmark45)  [31](#_bookmark49)[,35](#_bookmark53)[,39](#_bookmark57)[,41](#_bookmark59)[,43,](#_bookmark61)  [53](#_bookmark71)[,54](#_bookmark72) |  |
|  | ✔ | 4. Determine if regulatory approval is a requirement or not for considering interventions (e.g. for international guidelines this may be not relevant as regulatory approval may not be present for all target countries). | [4](#_bookmark22)[,5](#_bookmark23)[,7](#_bookmark25)[,8](#_bookmark26)[,14](#_bookmark32)[,21](#_bookmark39)[,24,](#_bookmark42)  [31](#_bookmark49)[,32](#_bookmark50) |  |
|  | ✔ | 5. Explicitly describe the intervention(s) and comparator(s) to be considered in the guideline and develop an analytic framework depicting the relationships among interventions and outcomes. Identify whether or not  multiple (treatment) comparisons should be included. | [4-9](#_bookmark22)[,11-16](#_bookmark29)[,19-21,](#_bookmark37)  [23](#_bookmark41)[,24](#_bookmark42)[,34](#_bookmark52)[,35](#_bookmark53)[,43,](#_bookmark61)  [46](#_bookmark64)[,55](#_bookmark73) |  |
|  | ✔ | 6. Identify the important outcomes (e.g. outcomes along the clinical pathway; morbidity, quality of life, mortality), including both desirable (e.g. benefits, less burden, savings) and undesirable effects (e.g. harm, burden, costs, and decrease in patient autonomy). Do not ignore important outcomes for which evidence may be lacking, | [4-9](#_bookmark22)[,11-16](#_bookmark29)[,19-21,](#_bookmark37)  [23](#_bookmark41)[,24](#_bookmark42)[,27](#_bookmark45)[,34](#_bookmark52)[,39,](#_bookmark57)  [43](#_bookmark61)[,46](#_bookmark64)[,52](#_bookmark70) |  |
|  | ✔ | 7. Determine the setting (e.g. countries, hospitals) or include it in the considerations about the population (i.e. population cared for in tertiary care hospitals). | [4-7](#_bookmark22)[,19](#_bookmark37)[,20,](#_bookmark38)  [24](#_bookmark42)[,34](#_bookmark52)[,45](#_bookmark63)[,52](#_bookmark70) |  |
|  | ✔ | 8. Mandate a preference for patient-important outcomes over surrogate, indirect outcomes. Consider appropriateness of surrogate outcomes along the causal pathway when data for a patient-important outcome is lacking. | [4-6](#_bookmark22)[,14](#_bookmark32)[,16,](#_bookmark34)  [19](#_bookmark37)[,20](#_bookmark38)[,24](#_bookmark42)[,34](#_bookmark52)[,39,](#_bookmark57)  [43](#_bookmark61)[,46](#_bookmark64)[,52](#_bookmark70) |  |

|  | ✔ | 9. Rank the relative importance of the outcomes, taking into consideration the values and preferences of the target population. | [4](#_bookmark22)[,5](#_bookmark23)[,7](#_bookmark25)[,11-16](#_bookmark29)[,20,](#_bookmark38)  [24](#_bookmark42)[,34](#_bookmark52)[,43](#_bookmark61)[,52](#_bookmark70) |  |
| --- | --- | --- | --- | --- |
|  | ✔ | 10. Determine or develop a process for determining *a priori* the magnitude of effect for the individual outcomes that is judged as important to the target population. | [34](#_bookmark52)[,43](#_bookmark61) |  |
|  | ✔ | 11. Involve all guideline group members and consult consumers and stakeholders to ensure broad representation from the target population in generating the questions and selecting and rating the important  outcomes. | [1](#_bookmark19)[,4](#_bookmark22)[,5](#_bookmark23)[,7](#_bookmark25)[,14](#_bookmark32)[,20](#_bookmark38)[,24,](#_bookmark42)  [32](#_bookmark50)[,52](#_bookmark70) |  |
|  | ✔ | 12. Document the methods of question generation and prioritization, selection and ranking of outcomes, and stakeholder and consumer consultation to ensure they are  explicit transparent. | [4](#_bookmark22)[,5](#_bookmark23)[,7](#_bookmark25)[,13](#_bookmark31)[,34](#_bookmark52)[,45](#_bookmark63) |  |
|  | ✔ | 13. Ensure the guideline protocol outlines the target population, target condition, outcomes, and key questions considered to help direct the evidence review. | [5](#_bookmark23)[,11](#_bookmark29)[,13](#_bookmark31)[,15](#_bookmark33)[,23,](#_bookmark41)  [27](#_bookmark45)[,34](#_bookmark52)[,35](#_bookmark53)[,43](#_bookmark61)[,45](#_bookmark63) |  |
| **[9. Considering Importance of Outcomes and Interventions, Values, Preferences and Utilities](#_bookmark0)** | | | | |
| ✔ |  | 1. Decide whether the relative importance of outcomes and interventions, values, preferences or utilities of consumers and stakeholders (e.g. patients and target audience) to inform decisions and deliberations during the guideline development will be elicited indirectly or directly (e.g. review of the published literature vs. consultation with  consumers). | [1](#_bookmark19)[,4](#_bookmark22)[,6](#_bookmark24)[,7](#_bookmark25)[,9](#_bookmark27)[,11](#_bookmark29)[,13,](#_bookmark31)  [15](#_bookmark33)[,16](#_bookmark34)[,20](#_bookmark38)[,21](#_bookmark39)[,23,](#_bookmark41)  [24](#_bookmark42)[,27](#_bookmark45)[,37](#_bookmark55)[,43](#_bookmark61)[,47,](#_bookmark65)  [48](#_bookmark66) |  |
| ✔ |  | 2. Establish methods for consultation with consumers and stakeholders to obtain information about the relative importance of outcomes and interventions, values, preferences or utilities (e.g. involvement of consumers on  guideline panel, surveys or focus groups with broader representation of consumers). | [6](#_bookmark24)[,15](#_bookmark33)[,16](#_bookmark34)[,24](#_bookmark42)[,37,](#_bookmark55)  [47](#_bookmark65)[,48](#_bookmark66) |  |
| ✔ |  | 3. Determine if a structured approach for assessing the confidence in the obtained importance ratings, values, preferences and utilities (i.e. quality of the evidence in  them) will be used. | E, [48](#_bookmark66) |  |

|  | ✔ | 4. Determine if modelling will be used to integrate the relative importance of outcomes and interventions, values, preferences or utilities and how modelling will be done. | E, [46](#_bookmark64)[,48](#_bookmark66) |  |
| --- | --- | --- | --- | --- |
| ✔ |  | 5. Determine whose perspective(s) will be considered when obtaining information about the relative importance of outcomes and interventions, values, preferences or utilities and when making decisions or formulating recommendations (e.g. patients, public, society, clinicians). | [4](#_bookmark22)[,20](#_bookmark38)[,37](#_bookmark55)[,47](#_bookmark65)[,48](#_bookmark66) |  |
| ✔ |  | 6. Consider and document approaches for dealing with conflicting relative importance ratings for outcomes and interventions, values, preferences or utilities (e.g. patient vs. carer, patient vs. public). | [15](#_bookmark33)[,20](#_bookmark38)[,43](#_bookmark61)[,47](#_bookmark65)[,48](#_bookmark66) |  |
| ✔ |  | 7. Document the methods of obtaining information about the relative importance of outcomes and interventions, values, preferences or utilities to ensure they are explicit and transparent. | [1](#_bookmark19)[,6](#_bookmark24)[,11](#_bookmark29)[,15](#_bookmark33)[,27](#_bookmark45)[,43,](#_bookmark61)  [47](#_bookmark65)[,48](#_bookmark66) |  |
|  | ✔ | 8. Document if ethical considerations, such as whether recommendations should give special consideration to certain patient groups or conditions (e.g. elderly, rare disease, those affected by health inequalities). | [47](#_bookmark65) |  |
|  | ✔ | 9. Decide how to consider ethical or moral values in making healthcare recommendations (e.g. by considering religious, social, or cultural convictions). | [56](#_bookmark74) |  |
| **[10. Deciding what Evidence to Include and Searching for Evidence](#_bookmark0)** | | | | |
| ✔ |  | 1. Follow systematic review methods (either full systematic reviews or rapid systematic reviews depending on the topic and organization’s framework) or provide a rationale for why this is not done. | [3-16](#_bookmark21)[,19-25](#_bookmark37)[,27,](#_bookmark45)  [39](#_bookmark57)[,43](#_bookmark61)[,46](#_bookmark64)[,57](#_bookmark75)[,58](#_bookmark76) |  |

| ✔ |  | 2. Develop a protocol for locating, selecting, and synthesizing the evidence (e.g. conduct a search for existing systematic reviews, new systematic review and grey literature search) and determine the types of evidence to include (e.g. databases searched, types of studies, inclusion and exclusion criteria, searching for specific studies on adverse effects or deciding to abstract information on adverse effects from studies on benefit). | [3-16](#_bookmark21)[,19-25](#_bookmark37)[,27,](#_bookmark45)  [34](#_bookmark52)[,35](#_bookmark53)[,39](#_bookmark57)[,43](#_bookmark61)[,46,](#_bookmark64)  [57](#_bookmark75) |  |
| --- | --- | --- | --- | --- |
| ✔ |  | 3. Decide who will develop the search strategies and perform searching and selection of evidence (e.g. working group of guideline development group, outsource to external agency, form a relationship between guideline development group and external agency to collaborate on development of the guideline). | [2](#_bookmark20)[,4-11](#_bookmark22)[,13,](#_bookmark31)  [15](#_bookmark33)[,24](#_bookmark42)[,43](#_bookmark61)[,57](#_bookmark75) |  |
| ✔ |  | 4. Critically appraise existing systematic review(s) selected to be included using a validated tool (e.g. AMSTAR) to ensure it is of adequate quality and appropriate for use in the guideline. | [4-7](#_bookmark22)[,9-12](#_bookmark27)[,14-16,](#_bookmark32)  [20](#_bookmark38)[,24](#_bookmark42)[,39](#_bookmark57)[,43](#_bookmark61)[,46,](#_bookmark64)  [57](#_bookmark75)[,58](#_bookmark76) |  |
|  | ✔ | 5. If an existing systematic review is updated or requires updating, determine how new evidence will be included and how those who conducted the review will be contacted and possibly involved in the update. | [3](#_bookmark21)[,7](#_bookmark25)[,20](#_bookmark38)[,58](#_bookmark76) |  |
| ✔ |  | 6. If a new systematic review is required, conduct an assessment to determine if adequate resources (e.g. time and funding) are available to conduct a full systematic review. | [7](#_bookmark25)[,14](#_bookmark32)[,20](#_bookmark38)[,57](#_bookmark75) |  |
| ✔ |  | 7. If resources are limited, consider applying a rapid assessment methodology and explicitly describe the methodology, noting important limitations, uncertainties, and the need and urgency to undertake a full systematic review. | [7](#_bookmark25)[,8](#_bookmark26)[,20](#_bookmark38)[,57](#_bookmark75) |  |

| ✔ |  | 8. Establish methods for identifying additional evidence and unpublished data (e.g. suggestions from guideline panel members, consulting with stakeholders). | [5](#_bookmark23)[,8](#_bookmark26)[,11](#_bookmark29)[,13](#_bookmark31)[,16](#_bookmark34)[,19,](#_bookmark37)  [34](#_bookmark52)[,57](#_bookmark75) |  |
| --- | --- | --- | --- | --- |
|  | ✔ | 9. Set a policy for handling expert input (i.e. expert opinion is not evidence *per se* and should not be used as evidence; rather, experience or observations that support expert opinions should be described, identified and, if possible, appraised in a systematic and transparent way, e.g. in the conceptual framework). | [8](#_bookmark26)[,10](#_bookmark28)[,11](#_bookmark29)[,16](#_bookmark34)[,24](#_bookmark42)[,55](#_bookmark73) |  |
| ✔ |  | 10. Document and publish the search and selection of evidence, judging eligibility, range of evidence included, and search strategies used to ensure the methods are explicit and transparent. | [3-5](#_bookmark21)[,8](#_bookmark26)[,11](#_bookmark29)[,13,](#_bookmark31)  [14](#_bookmark32)[,16](#_bookmark34)[,19-21,](#_bookmark37)  [23](#_bookmark41)[,27](#_bookmark45)[,35](#_bookmark53)[,58](#_bookmark76) |  |
| **[11. Summarizing Evidence and Considering Additional Information](#_bookmark0)** | | | | |
| ✔ |  | 1. Summarize the evidence using a concise summary (e.g. evidence table, evidence profile or summary of findings table) of the best available evidence for each important outcome, including diagnostic test accuracy, anticipated benefits, harms, resources (costs), the quality of evidence  rating, and a summary of the relative and absolute results/estimate of effect for each outcome. | [4-8](#_bookmark22)[,10-16](#_bookmark28)[,19-21,](#_bookmark37)  [24](#_bookmark42)[,27](#_bookmark45)[,35](#_bookmark53)[,39](#_bookmark57)[,43,](#_bookmark61)  [46](#_bookmark64)[,57](#_bookmark75)[,58](#_bookmark76) |  |
|  | ✔ | 2. Provide a summary of the additional information needed to inform recommendations (e.g. qualitative narrative summary, evidence table), including values and preferences, factors that might modify the expected effects, need (prevalence, baseline risk, or status), effects on equity, feasibility, and the availability of resources. | [3-7](#_bookmark21)[,10](#_bookmark28)[,11](#_bookmark29)[,13-15,](#_bookmark31)  [20](#_bookmark38)[,23](#_bookmark41)[,24](#_bookmark42)[,27](#_bookmark45)[,31,](#_bookmark49)  [43](#_bookmark61)[,45-47](#_bookmark63)[,54,](#_bookmark72)  [57](#_bookmark75)[,59-61](#_bookmark77) |  |
| ✔ |  | 3. Establish methods for obtaining information about resource use and cost (e.g. searching for existing economic evaluations, developing economic model, performing cost- effectiveness analysis). | [4](#_bookmark22)[,5](#_bookmark23)[,7](#_bookmark25)[,11](#_bookmark29)[,13-15,](#_bookmark31)  [19-21](#_bookmark37)[,23](#_bookmark41)[,24](#_bookmark42)[,27,](#_bookmark45)  [43](#_bookmark61)[,46](#_bookmark64)[,59](#_bookmark77)[,61](#_bookmark79) |  |

| ✔ |  | 4. Identify the costs, resource use, and, if applicable, cost- effectiveness and describe the nature of the costs (patient, community, society) (e.g. affordability considerations, estimates of resource use and acquisition costs weighed directly against evidence of benefits and harms of an intervention). | [4](#_bookmark22)[,5](#_bookmark23)[,7](#_bookmark25)[,11](#_bookmark29)[,13-15,](#_bookmark31)  [19-21](#_bookmark37)[,23](#_bookmark41)[,24](#_bookmark42)[,27,](#_bookmark45)  [43](#_bookmark61)[,46](#_bookmark64)[,59](#_bookmark77)[,61](#_bookmark79) |  |
| --- | --- | --- | --- | --- |
|  | ✔ | 5. Document the methods in which the additional information is to be incorporated with the synthesized evidence to ensure transparency (e.g. formal consensus on patient values, consensus on equity issues, formal economic analysis, consideration of disaggregated resource use data in a qualitative manner,). | [4](#_bookmark22)[,5](#_bookmark23)[,7](#_bookmark25)[,10](#_bookmark28)[,11,](#_bookmark29)  [13-15](#_bookmark31)[,19-21,](#_bookmark37)  [24](#_bookmark42)[,31](#_bookmark49)[,35](#_bookmark53)[,43](#_bookmark61)[,46,](#_bookmark64)  [47](#_bookmark65)[,57](#_bookmark75)[,61](#_bookmark79) |  |
|  | ✔ | 6. Provide training about the use of the evidence tables and opportunities for discussion to ensure all members of the guideline panel are familiar with the tables and use them in the appropriate manner. | [2](#_bookmark20)[,15](#_bookmark33)[,28](#_bookmark46) |  |
|  | ✔ | 7. In addition to the evidence summary, make available the full systematic review(s) and the original studies and other sources of evidence for the guideline panel to inform deliberations (e.g. set up a collaborative website and/or make available at meetings and via electronic communication). | [15](#_bookmark33)[,57](#_bookmark75) |  |
| **[12. Judging Quality, Strength or Certainty of a Body of Evidence](#_bookmark0)** | | | | |
|  | ✔ | 1. Select a framework outlining the criteria to be considered in rating the quality of evidence (e.g. GRADE, USPSTF). Avoid modifying grading tools. | [4-16](#_bookmark22)[,19-25,](#_bookmark37)  [43](#_bookmark61)[,46](#_bookmark64)[,58](#_bookmark76)[,62](#_bookmark80) |  |
|  | ✔ | 2. Decide who will be responsible for appraising the quality of evidence (e.g. un-conflicted methodologists participating in the working group). | [4](#_bookmark22)[,6](#_bookmark24)[,10](#_bookmark28)[,14-16](#_bookmark32)[,63](#_bookmark81) |  |
|  | ✔ | 3. Assess the quality of evidence for each important outcome. | [4](#_bookmark22)[,5](#_bookmark23)[,7](#_bookmark25)[,11-16](#_bookmark29)[,43,](#_bookmark61)  [46](#_bookmark64)[,58](#_bookmark76)[,62](#_bookmark80) |  |

|  | ✔ | 4. Assess the overall quality of evidence (e.g. lowest quality of evidence from outcomes rated as most important or critical, or highest quality of evidence when all outcomes point in the same direction). | [4](#_bookmark22)[,5](#_bookmark23)[,7-9](#_bookmark25)[,11-16,](#_bookmark29)  [19-21](#_bookmark37)[,43](#_bookmark61)[,46,](#_bookmark64)  [58](#_bookmark76)[,62](#_bookmark80) |  |
| --- | --- | --- | --- | --- |
|  | ✔ | 5. Report the quality of evidence assessed for the outcomes and the body of evidence. | [4](#_bookmark22)[,7](#_bookmark25)[,8](#_bookmark26)[,12](#_bookmark30)[,14](#_bookmark32)[,15,](#_bookmark33)  [20](#_bookmark38)[,21](#_bookmark39)[,23](#_bookmark41)[,46](#_bookmark64)[,51,](#_bookmark69)  [63](#_bookmark81) |  |
|  | ✔ | 6. Document the judgements made in appraising the quality of evidence to ensure they are transparent and explicit. | [4](#_bookmark22)[,5](#_bookmark23)[,7](#_bookmark25)[,8](#_bookmark26)[,11](#_bookmark29)[,13-16,](#_bookmark31)  [19-21](#_bookmark37)[,23](#_bookmark41)[,43,](#_bookmark61)  [58](#_bookmark76)[,62](#_bookmark80) |  |
| **[13. Developing Recommendations and Determining their Strength](#_bookmark0)** | | | | |
| ✔ |  | 1. Apply a framework outlining the factors to be considered to arrive at a recommendation. | [3](#_bookmark21)[,5-16](#_bookmark23)[,19-21,](#_bookmark37)  [24](#_bookmark42)[,27](#_bookmark45)[,35](#_bookmark53)[,39](#_bookmark57)[,43,](#_bookmark61)  [46](#_bookmark64)[,62](#_bookmark80)[,64](#_bookmark82) |  |
| ✔ |  | 2. Plan and share the logistical details of the consensus meeting(s) during which recommendations will be formulated with the participants, including distribution of documents required for the meeting (e.g. evidence summaries, evidence-to-recommendation tables), setting an agenda for the meeting(s) and selecting a consensus development method to be used by the group in agreeing  on judgements (e.g. Delphi method, nominal group technique). | [7](#_bookmark25)[,9](#_bookmark27)[,10](#_bookmark28)[,16](#_bookmark34)[,19](#_bookmark37)[,43](#_bookmark61) |  |
| ✔ |  | 3. Review the factors of the framework that influence the recommendation, including the direction and strength (e.g. the types of evidence and information relevant to the analysis focusing on the balance between desirable and undesirable consequences informed by the quality of evidence, magnitude of the difference between the benefits and harms, the certainty about or variability in values and  preferences, resource use, equity and other factors). | [3-8](#_bookmark21)[,11](#_bookmark29)[,12](#_bookmark30)[,14-16,](#_bookmark32)  [19](#_bookmark37)[,20](#_bookmark38)[,23](#_bookmark41)[,24](#_bookmark42)[,27,](#_bookmark45)  [35](#_bookmark53)[,39](#_bookmark57)[,43](#_bookmark61)[,46](#_bookmark64)[,54,](#_bookmark72)  [62](#_bookmark80)[,64](#_bookmark82) |  |

| ✔ |  | 4. If applicable, make provisions for formulating recommendations in situations where there is insufficient evidence or very low quality evidence (e.g. conditional recommendation with judgements laid out transparently, no recommendation if the guideline panel feels there is substantial risk that their decision may be wrong, recommend that the intervention be used in the context of research complemented by guidance for what are the best management options until further research becomes available). | [4](#_bookmark22)[,5](#_bookmark23)[,7](#_bookmark25)[,11](#_bookmark29)[,13-15,](#_bookmark31)  [20](#_bookmark38)[,46](#_bookmark64)[,64](#_bookmark82) |  |
| --- | --- | --- | --- | --- |
| ✔ |  | 5. Make provisions for formulating research recommendations and decide where to report them (e.g. in the guideline appendix, suggesting the specific research questions, specific patient-important outcomes to measure and other relevant aspects of what research is needed to reduce the uncertainty about the benefits and/or undesirable downsides of the intervention). | [5-7](#_bookmark23)[,14](#_bookmark32)[,15,](#_bookmark33)  [46](#_bookmark64)[,64](#_bookmark82)[,65](#_bookmark83) |  |
| ✔ |  | 6. Formulate the recommendations and summarize the rationale for each recommendation (e.g. narratively or in a table), including details about the judgements made by the group and the explicit link between the recommendation and evidence supporting the recommendation. | [4-7](#_bookmark22)[,11-13](#_bookmark29)[,15,](#_bookmark33)  [16](#_bookmark34)[,20](#_bookmark38)[,21](#_bookmark39)[,24](#_bookmark42)[,27,](#_bookmark45)  [35](#_bookmark53)[,39](#_bookmark57)[,46](#_bookmark64)[,51](#_bookmark69)[,63,](#_bookmark81)  [64](#_bookmark82) |  |
| ✔ |  | 7. Select a method for rating the strength of the formulated recommendations to inform the audience of the guideline about the degree of the guideline group’s confidence about following that recommendation. | [4](#_bookmark22)[,6-9](#_bookmark24)[,12-16,](#_bookmark30)  [19-24](#_bookmark37)[,43](#_bookmark61)[,46,](#_bookmark64)  [62](#_bookmark80)[,64](#_bookmark82) |  |
| ✔ |  | 8. Select a consensus development method to be used by the group in rating the strength of recommendations (e.g.  Delphi method, nominal group technique, voting). | [2](#_bookmark20)[,4](#_bookmark22)[,6](#_bookmark24)[,7](#_bookmark25)[,15](#_bookmark33)[,16,](#_bookmark34)  [20](#_bookmark38)[,43](#_bookmark61) |  |

| ✔ |  | 9. Provide suggestions about whether the recommendations are appropriate to serve as performance measures/quality criteria (e.g. management options associated with strong recommendations based on high- or moderate-quality evidence are particularly good candidates for quality criteria, when a recommendation is weak, discussing with patients the relative merits of the alternative management strategies and appropriate documentation of this interaction may become a quality criterion). | [4](#_bookmark22)[,9](#_bookmark27)[,13](#_bookmark31)[,16](#_bookmark34)[,24](#_bookmark42)[,41,](#_bookmark59)  [64](#_bookmark82) |  |
| --- | --- | --- | --- | --- |
| ✔ |  | 10. Document the judgements made in formulating the recommendations and determining their strength to ensure they are transparent and explicit. | [3](#_bookmark21)[,4](#_bookmark22)[,6-8](#_bookmark24)[,11-13,](#_bookmark29)  [16](#_bookmark34)[,19-21](#_bookmark37)[,23](#_bookmark41)[,24,](#_bookmark42)  [27](#_bookmark45)[,35](#_bookmark53)[,43](#_bookmark61)[,51](#_bookmark69)[,64](#_bookmark82) |  |
| **[14. Wording of Recommendations and of Considerations of Implementation, Feasibility and Equity](#_bookmark0)** | | | | |
| ✔ |  | 1. Decide on standardized wording to use for recommendation statements to ensure clarity and to maintain consistency throughout the guideline, avoiding wording that may be vague and nonspecific. | [4](#_bookmark22)[,5](#_bookmark23)[,7-9](#_bookmark25)[,13](#_bookmark31)[,14,](#_bookmark32)  [16](#_bookmark34)[,19-21](#_bookmark37)[,23,](#_bookmark41)  [24](#_bookmark42)[,27](#_bookmark45)[,43](#_bookmark61)[,46](#_bookmark64)[,64](#_bookmark82) |  |
| ✔ |  | 2. Write the recommendations in a way that is actionable with sufficient information so that it is not necessary for guideline users to refer to other material in order to understand the recommendation. | [4](#_bookmark22)[,5](#_bookmark23)[,7-9](#_bookmark25)[,14](#_bookmark32)[,16,](#_bookmark34)  [20](#_bookmark38)[,21](#_bookmark39)[,23](#_bookmark41)[,24](#_bookmark42)[,27,](#_bookmark45)  [35](#_bookmark53)[,46](#_bookmark64)[,51](#_bookmark69)[,63](#_bookmark81) |  |
| ✔ |  | 3. Provide clear direction or an interpretation aid to describe the implication of the strength of recommendation for clinicians, patients, policy makers, and any other target audience groups. | [4](#_bookmark22)[,8](#_bookmark26)[,9](#_bookmark27)[,13](#_bookmark31)[,14](#_bookmark32)[,16,](#_bookmark34)  [19](#_bookmark37)[,20](#_bookmark38)[,35](#_bookmark53)[,46](#_bookmark64)[,64](#_bookmark82) |  |
| ✔ |  | 4. Indicate in the recommendation statements the population for which the recommendation is intended, the intervention being recommended, and the alternative approach(es) or intervention(s). | [4](#_bookmark22)[,5](#_bookmark23)[,8](#_bookmark26)[,9](#_bookmark27)[,13](#_bookmark31)[,16](#_bookmark34)[,20,](#_bookmark38)  [35](#_bookmark53)[,46](#_bookmark64)[,63](#_bookmark81)[,64](#_bookmark82) |  |
| ✔ |  | 5. Include remarks that describe the context, feasibility and applicability of the recommendation and highlight key considerations such as equity issues and specific conditions that might apply to the recommendation (e.g. whether the conditions outlined apply to a specific subpopulation,  specific types of the intervention, for certain values and preferences, when certain resources are available, etc.). | [3-5](#_bookmark21)[,8](#_bookmark26)[,9](#_bookmark27)[,13](#_bookmark31)[,16,](#_bookmark34)  [20](#_bookmark38)[,24](#_bookmark42)[,31](#_bookmark49)[,35](#_bookmark53)[,43,](#_bookmark61)  [46](#_bookmark64)[,48](#_bookmark66)[,54](#_bookmark72)[,60](#_bookmark78)[,64](#_bookmark82) |  |

| ✔ |  | 6. Report the quality of evidence and the strength of recommendation in proximity to the recommendation statement. | [6-9](#_bookmark24)[,13](#_bookmark31)[,16](#_bookmark34)[,19-21,](#_bookmark37)  [24](#_bookmark42)[,35](#_bookmark53)[,46](#_bookmark64)[,51](#_bookmark69) |  |
| --- | --- | --- | --- | --- |
| ✔ |  | 7. Establish methods to be used by the group in agreeing on the final wording of recommendation statements (e.g. review and approval, formal consensus). | [4](#_bookmark22)[,7](#_bookmark25)[,9](#_bookmark27)[,16](#_bookmark34)[,21,](#_bookmark39)  [43](#_bookmark61)[,64](#_bookmark82) |  |
| ✔ |  | 8. Report the recommendations in a way that is comprehensible and visible (e.g. do not embed recommendations within long paragraphs, group  recommendations together in a summary section). | [5](#_bookmark23)[,9](#_bookmark27)[,14-16](#_bookmark32)[,24](#_bookmark42)[,27,](#_bookmark45)  [46](#_bookmark64)[,63](#_bookmark81) |  |
| **[15. Reporting and Peer Review](#_bookmark0)** | | | | |
| ✔ |  | 1. Develop or adopt a standardized format for reporting the guideline, with specific structure, headings, and content. | [5-7](#_bookmark23)[,9-11](#_bookmark27)[,14-16,](#_bookmark32)  [18](#_bookmark36)[,20-22](#_bookmark38)[,25](#_bookmark43)[,35,](#_bookmark53)  [39](#_bookmark57)[,43](#_bookmark61)[,51](#_bookmark69)[,63](#_bookmark81) |  |
| ✔ |  | 2. Decide on the format(s) to be prepared for the guideline product(s) (e.g. full guideline, full guideline with technical report/systematic reviews, brief guideline for clinicians or policymakers, consumer version for patients) that will correspond to the dissemination plan. (*see Topic* [*16*](#_bookmark16)) | [3-12](#_bookmark21)[,14-16,](#_bookmark32)  [20-22](#_bookmark38)[,24](#_bookmark42)[,25](#_bookmark43)[,41,](#_bookmark59)  [51](#_bookmark69)[,63](#_bookmark81) |  |
| ✔ |  | 3. Decide who will be responsible for writing the guideline product(s) (e.g. sub-committee of the guideline working group) and decide on authorship (e.g. individual authors, organization as author, working group as author). (*see Topic 1*) | [3-5](#_bookmark21)[,7](#_bookmark25)[,8](#_bookmark26)[,11,](#_bookmark29)  [13-16](#_bookmark31)[,20](#_bookmark38)[,43](#_bookmark61)[,63](#_bookmark81) |  |
| ✔ |  | 4. Conduct a review of the final draft of the guideline report(s) by all members of the guideline development group, allowing sufficient opportunity for feedback, editing and revisions. | [5-8](#_bookmark23)[,13](#_bookmark31)[,16](#_bookmark34)[,43,](#_bookmark61)  [63](#_bookmark81)[,65](#_bookmark83) |  |
| ✔ |  | 5. Seek approval from all members of the guideline development group for the final document(s). | [1](#_bookmark19)[,5](#_bookmark23)[,7-9](#_bookmark25)[,11](#_bookmark29)[,12,](#_bookmark30)  [16](#_bookmark34)[,21](#_bookmark39)[,24](#_bookmark42)[,43](#_bookmark61) |  |

| ✔ |  | 6. Initiate organizational (i.e. internal) peer review. | [5-13](#_bookmark23)[,15](#_bookmark33)[,16,](#_bookmark34)  [20](#_bookmark38)[,25](#_bookmark43)[,41](#_bookmark59)[,43,](#_bookmark61)  [63](#_bookmark81)[,65](#_bookmark83) |  |
| --- | --- | --- | --- | --- |
| ✔ |  | 7. Decide on the method(s) of external peer review, to review the final document(s) for accuracy, practicality, clarity, organization, and usefulness of the recommendations, as well as to ensure input from broader and important perspectives that the guideline group did not encompass (e.g. invited peer review, public consultation period with incorporation of feedback and responses from the guideline development group, submitting to peer-reviewed  publication). | [3](#_bookmark21)[,5-11](#_bookmark23)[,13-16](#_bookmark31)[,20-](#_bookmark38)  [25](#_bookmark38)[,27](#_bookmark45)[,28](#_bookmark46)[,39](#_bookmark57)[,41,](#_bookmark59)  [43](#_bookmark61)[,63](#_bookmark81)[,65](#_bookmark83) |  |
| ✔ |  | 8. Document the internal and external peer review process and, if applicable, publish consultation comments and the guideline development group’s responses. | [5-10](#_bookmark23)[,13](#_bookmark31)[,16,](#_bookmark34)  [21](#_bookmark39)[,23](#_bookmark41)[,25](#_bookmark43)[,35,](#_bookmark53)  [41](#_bookmark59)[,63](#_bookmark81)[,65](#_bookmark83) |  |
| **[16. Dissemination and Implementation](#_bookmark0)** | | | | |
| ✔ |  | 1. Prepare an active dissemination plan with various approaches to enhance the adoption of the guideline, taking into consideration utilization and copyrights (e.g. make guideline available online, develop formal relationships with those in health care systems responsible for guideline dissemination and implementation to support guideline uptake, press conference, social media strategy, dissemination at professional society meetings, publish guideline in a journal that is accessed by the target audience). | [3-5](#_bookmark21)[,7](#_bookmark25)[,9-12,](#_bookmark27)  [14-16](#_bookmark32)[,19-22](#_bookmark37)[,24,](#_bookmark42)  [39](#_bookmark57)[,41](#_bookmark59)[,43](#_bookmark61)[,66](#_bookmark84)[,67](#_bookmark85) |  |
| ✔ |  | 2. Develop or adapt tools, support, and derivative products to provide guidance on how the recommendations can be implemented into practice (e.g. mobile applications, integration with clinical decision support systems, make guideline adaptable as an educational resource for target audience for education outreach). | [4-8](#_bookmark22)[,11](#_bookmark29)[,12](#_bookmark30)[,15,](#_bookmark33)  [16](#_bookmark34)[,20](#_bookmark38)[,21](#_bookmark39)[,24](#_bookmark42)[,25,](#_bookmark43)  [27](#_bookmark45)[,35](#_bookmark53)[,41](#_bookmark59)[,43,](#_bookmark61)  [60](#_bookmark78)[,63](#_bookmark81) |  |

| ✔ |  | 3. Make considerations for adaptation of the guideline and provide specific instructions for how target end users who would like to adapt the guidelines to other contexts can do so in a systematic and transparent way (e.g. modifying a recommendation based on local resources and baseline risk, implications that deviate from the judgements made by the guideline panel). | [7](#_bookmark25)[,15](#_bookmark33)[,16](#_bookmark34)[,19](#_bookmark37)[,21,](#_bookmark39)  [27](#_bookmark45)[,38](#_bookmark56)[,60](#_bookmark78) |  |
| --- | --- | --- | --- | --- |
|  | ✔ | 4. Set rules and regulations for translation of the guideline into other languages (e.g. allow translation by third party organizations following approval by the guideline group, include staff responsible for translation in guideline working group). | [7](#_bookmark25)[,16](#_bookmark34)[,25](#_bookmark43)[,38](#_bookmark56) |  |
| **[17. Evaluation and Use](#_bookmark0)** | | | | |
| ✔ |  | 1. Conduct an internal evaluation (i.e. self-assessment) of the guideline development process, including the guideline panel meeting(s) held to formulate recommendations, by asking guideline group members for feedback. | E, [65](#_bookmark83) |  |
| ✔ |  | 2. Consider pilot testing the guideline with the target end users (e.g. with members of target audience and stakeholders who participated in the guideline development group). | [6](#_bookmark24)[,15](#_bookmark33)[,16](#_bookmark34)[,19](#_bookmark37)[,27,](#_bookmark45)  [35](#_bookmark53)[,65](#_bookmark83) |  |
| ✔ |  | 3. Provide criteria and tools for target end users to monitor and audit the implementation and use of the guideline recommendations (e.g. identify outcomes that should change with implementation and suggest methods for measuring the outcomes). | [3-7](#_bookmark21)[,9](#_bookmark27)[,11](#_bookmark29)[,12,](#_bookmark30)  [14-16](#_bookmark32)[,19](#_bookmark37)[,21](#_bookmark39)[,24,](#_bookmark42)  [27](#_bookmark45)[,35](#_bookmark53)[,38](#_bookmark56)[,41](#_bookmark59)[,65](#_bookmark83) |  |
| ✔ |  | 4. Provide support and tools for prospective evaluation of the guideline to determine its effectiveness after implementation (e.g. using randomized evaluations where possible, using before-after evaluations cautiously due to  uncertainties regarding the effects of implementation). | [4](#_bookmark22)[,5](#_bookmark23)[,7](#_bookmark25)[,11](#_bookmark29)[,15](#_bookmark33)[,19,](#_bookmark37)  [21](#_bookmark39)[,65](#_bookmark83)[,66](#_bookmark84) |  |
| ✔ |  | 5. Consider the potential involvement of the guideline development group in prospective evaluation(s) of the guideline (e.g. partnering with organizations that implement the guideline to plan evaluation studies). | [5](#_bookmark23)[,7](#_bookmark25)[,15](#_bookmark33)[,16](#_bookmark34)[,24](#_bookmark42)[,25,](#_bookmark43)  [65](#_bookmark83)[,66](#_bookmark84) |  |

| ✔ |  | 6. Plan to collect feedback and evaluations from users to identify how to improve the intrinsic implementability of the recommendations in subsequent versions of the guideline. | [4-6](#_bookmark22)[,11](#_bookmark29)[,16,](#_bookmark34)  [19](#_bookmark37)[,24](#_bookmark42)[,38](#_bookmark56)[,65](#_bookmark83) |  |
| --- | --- | --- | --- | --- |
| **[18. Updating](#_bookmark0)** | | | | |
| ✔ |  | 1. Set a policy, procedure and timeline for routinely monitoring and reviewing whether the guideline needs to be updated (e.g. update systematic review every 3 years to determine if there is any new evidence available). | [3-9](#_bookmark21)[,11](#_bookmark29)[,12,](#_bookmark30)  [14-16](#_bookmark32)[,19-25](#_bookmark37)[,27,](#_bookmark45)  [35](#_bookmark53)[,38](#_bookmark56)[,41](#_bookmark59)[,43,](#_bookmark61)  [65](#_bookmark83)[,68](#_bookmark86) |  |
|  | ✔ | 2. Decide who will be responsible for routinely monitoring the literature and assessing whether new significant evidence is available (e.g. consider involvement of experts not previously involved in the guideline development group to periodically review the guideline). | [3](#_bookmark21)[,5-9](#_bookmark23)[,14-16](#_bookmark32)[,20,](#_bookmark38)  [24](#_bookmark42)[,38](#_bookmark56)[,43](#_bookmark61)[,65](#_bookmark83) |  |
|  | ✔ | 3. Set the conditions that will determine when a partial or a full update of the guideline is required (e.g. if only certain recommendation statements need to be updated, or whether many recommendations are out of date making the entire guideline invalid, or when recommendations are necessary for newly available treatments). | [3-7](#_bookmark21)[,9](#_bookmark27)[,11](#_bookmark29)[,15,](#_bookmark33)  [16](#_bookmark34)[,20](#_bookmark38)[,22-24,](#_bookmark40)  [41](#_bookmark59)[,65](#_bookmark83)[,68](#_bookmark86) |  |
|  | ✔ | 4. Make arrangements for guideline group membership and participation after completion of the guideline (e.g. rotating membership every 1-2 years, selection of a new group at time of updating, continuing participation by guideline panel chair). | [5](#_bookmark23)[,9](#_bookmark27)[,13](#_bookmark31)[,20](#_bookmark38)[,25](#_bookmark43)[,38,](#_bookmark56)  [65](#_bookmark83) |  |
|  | ✔ | 5. Plan the funding and logistics for updating the guideline in the future (e.g. securing ongoing funding, standing  oversight committee to oversee the updating process). | [15](#_bookmark33)[,16](#_bookmark34)[,65](#_bookmark83) |  |
|  | ✔ | 6. Document the plan and proposed methods for updating the guideline to ensure they are followed. | [3](#_bookmark21)[,15](#_bookmark33)[,16](#_bookmark34)[,27](#_bookmark45)[,35](#_bookmark53)[,68](#_bookmark86) |  |

*E – Item informed by expert consultation

# REFERENCES

1. Fretheim A, Schunemann H, Oxman A. Improving the use of research evidence in guideline development: 3. Group composition and consultation process. *Health Research Policy and Systems.* 2006;4(1):15.
2. Kunz R, Fretheim A, Cluzeau F, et al. Guideline Group Composition and Group Processes: Article 3 in Integrating and Coordinating Efforts in COPD Guideline Development. An Official ATS/ERS Workshop Report. *Proceedings of the American Thoracic Society.* 2012;9(5):229-233.
3. The ADAPTE Collaboration. The ADAPTE Process: Resource Toolkit for Guideline Adaptation. Version 2.0. 2009; [http://www.g-i-](http://www.g-i-n.net/document-store/working-groups-documents/adaptation/adapte-resource-toolkit-guideline-adaptation-2-0.pdf) [n.net/document-store/working-groups-documents/adaptation/adapte-resource-toolkit-guideline-adaptation-2-0.pdf.](http://www.g-i-n.net/document-store/working-groups-documents/adaptation/adapte-resource-toolkit-guideline-adaptation-2-0.pdf) Accessed July 5, 2013.
4. World Health Organization. Estonian Handbook for Guidelines Development. 2011; [http://whqlibdoc.who.int/publications/2011/9789241502429_eng.pdf.](http://whqlibdoc.who.int/publications/2011/9789241502429_eng.pdf) Accessed April 22, 2013.
5. National Institute for Health and Clinical Excellence. The guidelines manual. 2012; [http://publications.nice.org.uk/the-guidelines-](http://publications.nice.org.uk/the-guidelines-manual-pmg6) [manual-pmg6.](http://publications.nice.org.uk/the-guidelines-manual-pmg6) Accessed April 22, 2013.
6. Scottish Intercollegiate Guidelines Network. SIGN 50: A guideline developer’s handbook. 2011; [http://www.sign.ac.uk/guidelines/fulltext/50/.](http://www.sign.ac.uk/guidelines/fulltext/50/) Accessed April 22, 2013.
7. World Health Organization. WHO Handbook for Guideline Development. 2012; [http://apps.who.int/iris/bitstream/10665/75146/1/9789241548441_eng.pdf.](http://apps.who.int/iris/bitstream/10665/75146/1/9789241548441_eng.pdf) Accessed April 22, 2013.
8. American College of Cardiology Foundation and American Heart Association. Methodology Manual and Policies from the ACCF/AHA Task Force on Practice Guidelines. 2010; [http://my.americanheart.org/professional/StatementsGuidelines/PoliciesDevelopment/Development/Methodologies-and-](http://my.americanheart.org/professional/StatementsGuidelines/PoliciesDevelopment/Development/Methodologies-and-Policies-from-the-ACCAHA-Task-Force-on-Practice-Guidelines_UCM_320470_Article.jsp) [Policies-from-the-ACCAHA-Task-Force-on-Practice-Guidelines_UCM_320470_Article.jsp.](http://my.americanheart.org/professional/StatementsGuidelines/PoliciesDevelopment/Development/Methodologies-and-Policies-from-the-ACCAHA-Task-Force-on-Practice-Guidelines_UCM_320470_Article.jsp) Accessed April 22, 2013.
9. Rosenfeld RM, Shiffman RN, Robertson P. Clinical Practice Guideline Development Manual, Third Edition: A Quality-Driven Approach for Translating Evidence into Action. *Otolaryngology -- Head and Neck Surgery.* 2013;148(1 suppl):S1-S55.
10. Cancer Care Ontario. Program in Evidence-Based Care Handbook. 2012; [http://www.cancercare.on.ca/about/programs/pebc/pebc-products/.](http://www.cancercare.on.ca/about/programs/pebc/pebc-products/) Accessed April 22, 2013.
11. Centers for Disease Control and Prevention. *Guidelines and Recommendations: A CDC Primer.* Atlanta, Georgia: Office of the Associate Director for Science Centers for Disease Control and Prevention;2012.
12. Davino-Ramaya C, Krause LK, Robbins CW, et al. Transparency matters: Kaiser Permanente's National Guideline Program methodological processes. *The Permanente journal.* Winter 2012;16(1):55-62.
13. Canadian Task Force on Preventive Health Care. Canadian Task Force on Preventive Health Care Procedure Manual. 2011; [http://canadiantaskforce.ca/methods/methods-manual/.](http://canadiantaskforce.ca/methods/methods-manual/) Accessed April 22, 2013.
14. Gutiérrez GC, Bossert T, Espinosa JQ, et al. Guía Metodológica para la elaboración de Guías de Atención Integral en el Sistema General de Seguridad Social en Salud Colombiano. 2010; [http://www.minsalud.gov.co/Documentos%20y%20Publicaciones/GUIA%20METODOLOGICA%20PARA%20LA%20ELABORACI](http://www.minsalud.gov.co/Documentos%20y%20Publicaciones/GUIA%20METODOLOGICA%20PARA%20LA%20ELABORACI%C3%93N%20DE%20GU%C3%8DAS%20DE%20ATENCI%C3%93N%20INTEGRAL.pdf)

[%C3%93N%20DE%20GU%C3%8DAS%20DE%20ATENCI%C3%93N%20INTEGRAL.pdf.](http://www.minsalud.gov.co/Documentos%20y%20Publicaciones/GUIA%20METODOLOGICA%20PARA%20LA%20ELABORACI%C3%93N%20DE%20GU%C3%8DAS%20DE%20ATENCI%C3%93N%20INTEGRAL.pdf) Accessed April 22, 2013.

1. Ministerio de Sanidad y Consumo. Elaboración de guías de práctica clínica en el sistema nacional de salud: Manual metodológico. 2007; [http://www.guiasalud.es/emanuales/elaboracion/index-02.html.](http://www.guiasalud.es/emanuales/elaboracion/index-02.html) Accessed April 22, 2013.
2. German Association of the Scientific Medical Societies (AWMF) - Standing Guidelines Commission. AWMF Guidance Manual and Rules for Guideline Development, 1st Edition, English Version. 2012; [http://www.awmf.org/leitlinien/awmf-regelwerk/awmf-](http://www.awmf.org/leitlinien/awmf-regelwerk/awmf-guidance.html) [guidance.html.](http://www.awmf.org/leitlinien/awmf-regelwerk/awmf-guidance.html) Accessed May 2, 2014.
3. Oxman A, Schunemann H, Fretheim A. Improving the use of research evidence in guideline development: 2. Priority setting. *Health Research Policy and Systems.* 2006;4(1):14.
4. American College of Cardiology Foundation and American Heart Association. Manuscript Development Process. 2010; [http://my.americanheart.org/professional/StatementsGuidelines/PoliciesDevelopment/Policies-](http://my.americanheart.org/professional/StatementsGuidelines/PoliciesDevelopment/Policies-Development_UCM_316897_Article.jsp) [Development_UCM_316897_Article.jsp.](http://my.americanheart.org/professional/StatementsGuidelines/PoliciesDevelopment/Policies-Development_UCM_316897_Article.jsp) Accessed April 22, 2013.
5. New Zealand Guidelines Group. *Handbook for the preparation of explicit evidence-based clinical practice guidelines.* Wellington: New Zealand Guidelines Group;2001.
6. Agency for Healthcare Research and Quality. U.S. Preventive Services Task Force Procedure Manual. 2008; [http://www.uspreventiveservicestaskforce.org/uspstf08/methods/procmanual.htm.](http://www.uspreventiveservicestaskforce.org/uspstf08/methods/procmanual.htm) Accessed April 22, 2013.
7. National Health and Medical Research Council. Procedures and requirements for meeting the 2011 NHMRC standard for clinical practice guidelines. 2011; [http://www.nhmrc.gov.au/guidelines/publications/cp133-and-cp133a.](http://www.nhmrc.gov.au/guidelines/publications/cp133-and-cp133a) Accessed April 22, 2013.
8. Ministerio de Salud. Norma técnica para la elaboración de guías de practica clinica. 2005; [http://bvs.minsa.gob.pe/local/MINSA/1176_DGSP196.pdf.](http://bvs.minsa.gob.pe/local/MINSA/1176_DGSP196.pdf) Accessed April 22, 2013.
9. Qaseem A, Forland F, Macbeth F, Ollenschlager G, Phillips S, van der Wees P. Guidelines International Network: Toward international standards for clinical practice guidelines. *Ann Intern Med.* 2012;156(7):525-531.
10. Institute of Medicine Committee on Standards for Developing Trustworthy Clinical Practice Guidelines. Clinical Practice Guidelines We Can Trust. 2011; [http://www.nap.edu/openbook.php?record_id=13058.](http://www.nap.edu/openbook.php?record_id=13058) Accessed April 22, 2013.
11. European Society of Cardiology. Recommendations for Guidelines Production. 2010; [http://www.escardio.org/guidelines-](http://www.escardio.org/guidelines-surveys/esc-guidelines/about/Pages/rules-writing.aspx) [surveys/esc-guidelines/about/Pages/rules-writing.aspx.](http://www.escardio.org/guidelines-surveys/esc-guidelines/about/Pages/rules-writing.aspx) Accessed April 22, 2013.
12. Boyd EA, Akl EA, Baumann M, et al. Guideline Funding and Conflicts of Interest: Article 4 in Integrating and Coordinating Efforts in COPD Guideline Development. An Official ATS/ERS Workshop Report. *Proceedings of the American Thoracic Society.* 2012;9(5):234-242.
13. Brouwers MC, Kho ME, Browman GP, et al. AGREE II: Advancing guideline development, reporting and evaluation in health care.

*Canadian Medical Association Journal.* 2010;182(18):E839-E842.

1. Schunemann H, Fretheim A, Oxman A. Improving the use of research evidence in guideline development: 1. Guidelines for guidelines. *Health Research Policy and Systems.* 2006;4(1):13.
2. National Institute for Health and Clinical Excellence. How NICE clinical guidelines are developed: An overview for stakeholders, the public and the NHS. 5th Edition. 2012; [http://publications.nice.org.uk/pmg6f.](http://publications.nice.org.uk/pmg6f) Accessed April 22, 2013.
3. Jacobs AK, Kushner FG, Ettinger SM, et al. ACCF/AHA clinical practice guideline methodology summit report: a report of the American College of Cardiology Foundation/American Heart Association Task Force on Practice Guidelines. *J Am Coll Cardiol.* 2013;61(2):213-265.
4. Oxman A, Schunemann H, Fretheim A. Improving the use of research evidence in guideline development: 12. Incorporating considerations of equity. *Health Research Policy and Systems.* 2006;4(1):24.
5. Cluzeau F, Wedzicha JA, Kelson M, et al. Stakeholder Involvement: How to Do It Right: Article 9 in Integrating and Coordinating Efforts in COPD Guideline Development. An Official ATS/ERS Workshop Report. *Proceedings of the American Thoracic Society.* 2012;9(5):269-273.
6. Fretheim A, Schunemann H, Oxman A. Improving the use of research evidence in guideline development: 5. Group processes.

*Health Research Policy and Systems.* 2006;4(1):17.

1. Wilt TJ, Guyatt G, Kunz R, et al. Deciding What Type of Evidence and Outcomes to Include in Guidelines: Article 5 in Integrating and Coordinating Efforts in COPD Guideline Development. An Official ATS/ERS Workshop Report. *Proceedings of the American Thoracic Society.* 2012;9(5):243-250.
2. Shiffman RN, Shekelle P, Overhage JM, Slutsky J, Grimshaw J, Deshpande AM. Standardized reporting of clinical practice guidelines: a proposal from the Conference on Guideline Standardization. *Ann Intern Med.* 2003;139(6):493-498.
3. Atkins D, Perez-Padilla R, MacNee W, Buist AS, Cruz AA. Priority Setting in Guideline Development: Article 2 in Integrating and Coordinating Efforts in COPD Guideline Development. An Official ATS/ERS Workshop Report. *Proceedings of the American Thoracic Society.* 2012;9(5):225-228.
4. Eccles M, Grimshaw J, Shekelle P, Schunemann H, Woolf S. Developing clinical practice guidelines: target audiences, identifying topics for guidelines, guideline group composition and functioning and conflicts of interest. *Implementation Science.* 2012;7(1):60.
5. Burgers JS, Anzueto A, Black PN, et al. Adaptation, Evaluation, and Updating of Guidelines: Article 14 in Integrating and Coordinating Efforts in COPD Guideline Development. An Official ATS/ERS Workshop Report. *Proceedings of the American Thoracic Society.* 2012;9(5):304-310.
6. Esandi ME, Luca MD, Chapman E, Schapochnik N, Bernztein R, Otheguy L. Guía para la adaptación de Guías de Práctica Clínica. 2008; [http://publicaciones.ops.org.ar/publicaciones/otras%20pub/GuiadeGuias.pdf.](http://publicaciones.ops.org.ar/publicaciones/otras%20pub/GuiadeGuias.pdf) Accessed April 22, 2013.
7. Schunemann HJ, Woodhead M, Anzueto A, et al. A guide to guidelines for professional societies and other developers of recommendations: Introduction to integrating and coordinating efforts in COPD guideline development. An official ATS/ERS workshop report. *Proceedings of the American Thoracic Society.* 2012;9(5):215-218.
8. Shekelle P, Woolf S, Grimshaw J, Schunemann H, Eccles M. Developing clinical practice guidelines: reviewing, reporting, and publishing guidelines; updating guidelines; and the emerging issues of enhancing guideline implementability and accounting for comorbid conditions in guideline development. *Implementation Science.* 2012;7(1):62.
9. Yawn BP, Akl EA, Qaseem A, Black P, Campos-Outcalt D. Identifying Target Audiences: Who Are the Guidelines For?: Article 1 in Integrating and Coordinating Efforts in COPD Guideline Development. An Official ATS/ERS Workshop Report. *Proceedings of the American Thoracic Society.* 2012;9(5):219-224.
10. Guyatt GH, Norris SL, Schulman S, et al. Methodology for the development of antithrombotic therapy and prevention of thrombosis guidelines: Antithrombotic Therapy and Prevention of Thrombosis, 9th ed: American College of Chest Physicians Evidence-Based Clinical Practice Guidelines. *Chest.* 2012;141(2 Suppl):53S-70S.
11. National Institute for Health and Clinical Excellence. The guidelines manual: Appendix A – Agreements and advice for Guideline Development Group members. 2012; [http://publications.nice.org.uk/pmg6a.](http://publications.nice.org.uk/pmg6a) Accessed April 22, 2013.
12. National Health and Medical Research Council. Guideline Development and Conflicts of Interest: Identifying and Managing Conflicts of Interest of Prospective Members and Members of NHMRC Committees and Working Groups Developing Guidelines. 2012; [http://www.nhmrc.gov.au/guidelines-and-publications/information-guideline-developers/guideline-development-and-](http://www.nhmrc.gov.au/guidelines-and-publications/information-guideline-developers/guideline-development-and-conflicts) [conflicts.](http://www.nhmrc.gov.au/guidelines-and-publications/information-guideline-developers/guideline-development-and-conflicts) Accessed April 22, 2013.
13. Woolf S, Schunemann H, Eccles M, Grimshaw J, Shekelle P. Developing clinical practice guidelines: types of evidence and outcomes; values and economics, synthesis, grading, and presentation and deriving recommendations. *Implementation Science.* 2012;7(1):61.
14. Kelson M, Akl EA, Bastian H, et al. Integrating Values and Consumer Involvement in Guidelines with the Patient at the Center: Article 8 in Integrating and Coordinating Efforts in COPD Guideline Development. An Official ATS/ERS Workshop Report. *Proceedings of the American Thoracic Society.* 2012;9(5):262-268.
15. Schunemann H, Fretheim A, Oxman A. Improving the use of research evidence in guideline development: 10. Integrating values and consumer involvement. *Health Research Policy and Systems.* 2006;4(1):22.
16. Boyd E, Bero L. Improving the use of research evidence in guideline development: 4. Managing conflicts of interests. *Health Research Policy and Systems.* 2006;4(1):16.
17. Cancer Care Ontario. Program in Evidence-Based Care Conflict of Interest Policy. 2011; [http://www.cancercare.on.ca/cms/one.aspx?objectId=7582&contextId=1377.](http://www.cancercare.on.ca/cms/one.aspx?objectId=7582&contextId=1377) Accessed April 22, 2013.
18. Oxman A, Schunemann H, Fretheim A. Improving the use of research evidence in guideline development: 14. Reporting guidelines.

*Health Research Policy and Systems.* 2006;4(1):26.

1. Schunemann H, Oxman A, Fretheim A. Improving the use of research evidence in guideline development: 6. Determining which outcomes are important. *Health Research Policy and Systems.* 2006;4(1):18.
2. Fabbri LM, Boyd C, Boschetto P, et al. How to Integrate Multiple Comorbidities in Guideline Development: Article 10 in Integrating and Coordinating Efforts in COPD Guideline Development. An Official ATS/ERS Workshop Report. *Proceedings of the American Thoracic Society.* 2012;9(5):274-281.
3. National Institute for Health and Clinical Excellence. Positively Equal: A guide to addressing equality issues in developing clinical guidelines. 2012; [http://www.nice.org.uk/aboutnice/howwework/developingniceclinicalguidelines/PositivelyEqual.jsp.](http://www.nice.org.uk/aboutnice/howwework/developingniceclinicalguidelines/PositivelyEqual.jsp) Accessed July 5, 2013.
4. Oxman A, Schunemann H, Fretheim A. Improving the use of research evidence in guideline development: 7. Deciding what evidence to include. *Health Research Policy and Systems.* 2006;4(1):19.
5. Hofmann B. Toward a procedure for integrating moral issues in health technology assessment. *Int J Technol Assess Health Care.*

Summer 2005;21(3):312-318.

1. Oxman A, Schunemann H, Fretheim A. Improving the use of research evidence in guideline development: 8. Synthesis and presentation of evidence. *Health Research Policy and Systems.* 2006;4(1):20.
2. Guyatt G, Akl EA, Oxman A, et al. Synthesis, Grading, and Presentation of Evidence in Guidelines: Article 7 in Integrating and Coordinating Efforts in COPD Guideline Development. An Official ATS/ERS Workshop Report. *Proceedings of the American Thoracic Society.* 2012;9(5):256-261.
3. Edejer T. Improving the use of research evidence in guideline development: 11. Incorporating considerations of cost-effectiveness, affordability and resource implications. *Health Research Policy and Systems.* 2006;4(1):23.
4. Schunemann H, Fretheim A, Oxman A. Improving the use of research evidence in guideline development: 13. Applicability, transferability and adaptation. *Health Research Policy and Systems.* 2006;4(1):25.
5. Hill SR, Olson LG, Falck-Ytter Y, et al. Incorporating Considerations of Cost-Effectiveness, Affordability, and Resource Implications in Guideline Development: Article 6 in Integrating and Coordinating Efforts in COPD Guideline Development. An Official ATS/ERS Workshop Report. *Proceedings of the American Thoracic Society.* 2012;9(5):251-255.
6. Schunemann H, Fretheim A, Oxman A. Improving the use of research evidence in guideline development: 9. Grading evidence and recommendations. *Health Research Policy and Systems.* 2006;4(1):21.
7. Wilson KC, Irwin RS, File TM, Schünemann HJ, Guyatt GH, Rabe KF. Reporting and Publishing Guidelines: Article 12 in Integrating and Coordinating Efforts in COPD Guideline Development. An Official ATS/ERS Workshop Report. *Proceedings of the American Thoracic Society.* 2012;9(5):293-297.
8. Schünemann HJ, Oxman AD, Akl EA, et al. Moving from Evidence to Developing Recommendations in Guidelines: Article 11 in Integrating and Coordinating Efforts in COPD Guideline Development. An Official ATS/ERS Workshop Report. *Proceedings of the American Thoracic Society.* 2012;9(5):282-292.
9. Oxman A, Schunemann H, Fretheim A. Improving the use of research evidence in guideline development: 16. Evaluation. *Health Research Policy and Systems.* 2006;4(1):28.
10. Fretheim A, Schunemann H, Oxman A. Improving the use of research evidence in guideline development: 15. Disseminating and implementing guidelines. *Health Research Policy and Systems.* 2006;4(1):27.
11. Grimshaw JM, Schünemann HJ, Burgers J, et al. Disseminating and Implementing Guidelines: Article 13 in Integrating and Coordinating Efforts in COPD Guideline Development. An Official ATS/ERS Workshop Report. *Proceedings of the American Thoracic Society.* 2012;9(5):298-303.
12. Cancer Care Ontario. Program in Evidence-Based Care Document Assessment and Review Protocol. 2012; [http://www.cancercare.on.ca/about/programs/pebc/document review/.](http://www.cancercare.on.ca/about/programs/pebc/document__review/) Accessed April 22, 2013.
