# Supplementary material for: Social Innovation For Health Research: Development of the SIFHR Checklist
Source: PLoS Med. 2021 Sep 13;18(9):e1003788. doi: 10.1371/journal.pmed.1003788 (PMC8475987; doi:10.1371/journal.pmed.1003788)
Supplement: S3 Text — (DOCX) [file pmed.1003788.s003.docx]

# **S3 Text: Delphi Survey Instrument**

Wednesday, July 8, 2020

**Social Innovation in Health Monitoring and Evaluation: Survey version 3**

Dear colleague,

Thank you for taking this social innovation in health monitoring and evaluation survey. This survey Will go through the monitoring and evaluation framework and then the research checklist. For each item, you will select one option about the extent to which you agree with this section being included in the framework/checklist. You will also have an opportunity to provide comments on each section. This survey will take approximately 20-25 minutes to complete, and we encourage you to complete and submit the survey in one attempt as answers will not be saved for completion and submission later. All responses will be strictly confidential, although we will collect some basic information at the start about socio- demographic characteristics.

We appreciate your help on this project!

#### Section A: Demographics

- 1. Gender
  2. Country
  3. Do you have any long-standing illness, disability, or infirmity? By long-standing I mean anything that has troubled you over a period of time or that is likely to affect you over a period of time. Y/N

#### Section B: The monitoring and Evaluation Framework

- 1. **Purpose:** The framework ties the social innovations in health with the impact and helps identify the set of activities required to logically progress towards achieving impact in short- and long- term scenarios. This framework aims to provide innovators, researchers and program managers with useful tools when designing, implementing, and/or evaluating social innovations in health as well as help individuals mitigate risks and improve their social innovations.

| **Strongly agree** | **Agree** | **Neutral** | **Disagree** | **Strongly disagree** |
| --- | --- | --- | --- | --- |
| **Comments:** | | | | |

- 1. **Definitions:** This framework defines a social innovation as a community-engaged process that links social change with health improvement, drawing on the diverse multi-disciplinary strengths of local individuals, communities, and institutions. Community is defined as people living in the same place or sharing common interests. We define stakeholders as end-users, community members, public sector officials, private sector leaders, community members, civil society, and other local individuals. Innovators are those developing the social innovation. End- users are direct beneficiaries of an innovation. Co-creation is a collaboration between innovators and end-users.

| **Strongly agree** | **Agree** | **Neutral** | **Disagree** | **Strongly disagree** |
| --- | --- | --- | --- | --- |
| **Comments:** | | | | |

- 1. **Process:** This framework was developed in partnership with the Social Innovation in Health Initiative (SIHI), a group convened by the UNICEF/UNDP/World Bank/WHO Special Programme for Research and Training in Tropical Diseases (TDR) and partners to advance social innovations globally. The framework involved a three-step process, including an open crowdsourcing call for ideas (described here), a scoping review, a series of multisectoral discussions and an adapted Delphi survey. This framework is intended for use in global settings, especially low- and middle- income countries. Our open access resources are in English, but you may contact your regional SIHI hub for more non-English resources in your region. The framework is divided into seven phases according to the Nesta seven stages for innovation. Working with end users (direct beneficiaries), co-creation is an essential part of social innovation in health and should be incorporated at each phase with an emphasis on the final phase in order to generate new institutional or social arrangements as well as develop a sustainable social innovation in health project. We have bolded ways where co-creation with end-users and key stakeholders can and should be incorporated at each phase of a project.

| **Strongly agree** | **Agree** | **Neutral** | **Disagree** | **Strongly disagree** |
| --- | --- | --- | --- | --- |
| **Comments:** | | | | |

#### Section C: Research Checklist

1. **Introduction**

This is a research checklist focused on social innovation in health. This checklist is relevant for researchers and implementers reporting social innovations in health and also reviewers for social innovation health projects. The checklist defines social innovation as a community-engaged process that links social change and health improvement, drawing on the diverse strengths of local individuals and institutions.

The purpose of this checklist is to provide researchers and reviewers a set of essential components to consider when reporting or reviewing a social innovation in health project. This is intended for researchers and innovators developing social innovations in health.

This checklist was developed in partnership with the Social Innovation in Health Initiative (SIHI), a group convened by the UNICEF/UNDP/World Bank/WHO Special Programme for Research and Training in Tropical Diseases and partners. Our group used a three- step process, including an open call for ideas, a scoping review, series of multisectoral discussions and an adapted Delphi. This is intended for use in global settings, especially low- and middle-income countries. The final research checklist was adapted from Tidier, a Template for Intervention Description and Replication Checklist.

| **Strongly agree** | **Agree** | **Neutral** | **Disagree** | **Strongly disagree** |
| --- | --- | --- | --- | --- |
| **Comments:** | | | | |

1. **BRIEF NAME:** The title or abstract identified this as a social innovation in health research study.

| **Strongly agree** | **Agree** | **Neutral** | **Disagree** | **Strongly disagree** |
| --- | --- | --- | --- | --- |
| **Comments:** | | | | |

1. **WHAT PROBLEM:** Described the problem addressed by the social innovation from the perspective of the end user.

| **Strongly agree** | **Agree** | **Neutral** | **Disagree** | **Strongly disagree** |
| --- | --- | --- | --- | --- |
| **Comments:** | | | | |

1. **WHAT RATIONALE:** Described the rationale and justification for the social innovation from the perspective of the end-user.

| **Strongly agree** | **Agree** | **Neutral** | **Disagree** | **Strongly disagree** |
| --- | --- | --- | --- | --- |
| **Comments:** | | | | |

1. **BENEFICIARIES:** Described the direct and indirect beneficiaries of the social innovation in health. Direct beneficiaries are the end users of the social innovation and indirect beneficiaries are others who also gain from the social innovation but are not end users. Described important characteristics of the direct beneficiaries.

| **Strongly agree** | **Agree** | **Neutral** | **Disagree** | **Strongly disagree** |
| --- | --- | --- | --- | --- |
| **Comments:** | | | | |

1. **STAKEHOLDER* INVOLVEMENT:** Described how local stakeholders were involved in study design, consultations, implementation, and evaluation of the social innovation in health

| **Strongly agree** | **Agree** | **Neutral** | **Disagree** | **Strongly disagree** |
| --- | --- | --- | --- | --- |
| **Comments:** | | | | |

1. **WHAT INPUTS**: Described any physical, digital or informational materials used or distributed during training, delivery and/or implementation of in the social innovation, provides information on where the materials can be accessed† (e.g. online, appendix, URL).

| **Strongly agree** | **Agree** | **Neutral** | **Disagree** | **Strongly disagree** |
| --- | --- | --- | --- | --- |
| **Comments:** | | | | |

1. **WHAT PROCESSES:** Described each of the processes, activities, and elements used in the social innovation, including any enabling or support activities. Described ethical approval if a research study.

| **Strongly agree** | **Agree** | **Neutral** | **Disagree** | **Strongly disagree** |
| --- | --- | --- | --- | --- |
| **Comments:** | | | | |

1. **WHO:** For each category of the social innovation **provider (e.g. trained layperson, nursing assistant, others), described their expertise, background, role and any specific training given?

| **Strongly agree** | **Agree** | **Neutral** | **Disagree** | **Strongly disagree** |
| --- | --- | --- | --- | --- |
| **Comments:** | | | | |

1. **HOW IMPLEMENTED:** Described the implementation strategy for the social innovation and whether it was delivered individually or in a group. Described the level of external resources for implementation (e.g., internet access).

| **Strongly agree** | **Agree** | **Neutral** | **Disagree** | **Strongly disagree** |
| --- | --- | --- | --- | --- |
| **Comments:** | | | | |

1. **WHERE:** Described the population, type(s) of location(s) where the social innovation is delivered, including any necessary social, political, cultural, environmental or other contextual issues. Described at what level the innovation is implemented (e.g., district, subdistrict, village).

| **Strongly agree** | **Agree** | **Neutral** | **Disagree** | **Strongly disagree** |
| --- | --- | --- | --- | --- |
| **Comments:** | | | | |

1. **HOW FREQUENT:** Described the frequency of the social innovation delivery and over what period of time including the number of sessions, their schedule, and their duration, intensity.

| **Strongly agree** | **Agree** | **Neutral** | **Disagree** | **Strongly disagree** |
| --- | --- | --- | --- | --- |
| **Comments:** | | | | |

1. **ADAPTABILITY:** Considered how the social innovation could be adapted for contexts other than the one described, if appropriate.

| **Strongly agree** | **Agree** | **Neutral** | **Disagree** | **Strongly disagree** |
| --- | --- | --- | --- | --- |
| **Comments:** | | | | |

1. **FINANCING:** Described how the social innovation in health was funded. Described how the social innovation could generate revenue (if applicable) or be institutionalized (if applicable) in order to be sustained in the future.

| **Strongly agree** | **Agree** | **Neutral** | **Disagree** | **Strongly disagree** |
| --- | --- | --- | --- | --- |
| **Comments:** | | | | |

1. **SOCIAL IMPACT:** Described the non-medial impact of the social innovation over a period of time. This could be impact on the environment, social changes, or other non-health impact (e.g. lessons learned, new processes that emerged from the project, new relationships and networks, application of learned processes to other problems). Described how the social impact was measured in the community.

| **Strongly agree** | **Agree** | **Neutral** | **Disagree** | **Strongly disagree** |
| --- | --- | --- | --- | --- |
| **Comments:** | | | | |

1. **HEALTH IMPACT**: Described the health impact of the social innovation over a period of time and the methods to assess health impact Health defined broadly here according to the WHO definition.

| **Strongly agree** | **Agree** | **Neutral** | **Disagree** | **Strongly disagree** |
| --- | --- | --- | --- | --- |
| **Comments:** | | | | |

1. **LIMITATIONS:** Described the limitations and potential unintended consequences of the social innovation in health during the design, development, or implementation.

| **Strongly agree** | **Agree** | **Neutral** | **Disagree** | **Strongly disagree** |
| --- | --- | --- | --- | --- |
| **Comments:** | | | | |

#### USEFUL RESOURCES

Introduction to Social Innovation in Health Initiative – Official website of SIHI- a global network of collaborators advancing Social Innovation in Health

Social Innovation in Health Case Studies – Cases studies and lessons learned from Low- and Middle-Income Countries

Social Innovation: The role of Research – A review and Policy Paper that examines the role of Social Innovation in Social Sciences and Humanities

TDR MOOC on Implementation Research –a six-week online training on implementation research (English, subtitles in French, Spanish)

Open Book of Social Innovation – A book on ways to design, develop and grow Social

| **Strongly agree** | **Agree** | **Neutral** | **Disagree** | **Strongly disagree** |
| --- | --- | --- | --- | --- |
| **Comments:** | | | | |

**THANK YOU FOR COMPLETING THIS SURVEY!**

i TDR, The Special Programme for Research and Training in Tropical Diseases. (2019). WHAT IS SOCIAL INNOVATION IN HEALTH? Retrieved from https://socialinnovationinhealth.org/about/what-is-social-innovation/

ii TDR, The Special Programme for Research and Training in Tropical Diseases. (2019). Riders for Health. Retrieved from https://socialinnovationinhealth.org/case-studies/riders-for-health-
