# Supplementary material for: Social Innovation For Health Research: Development of the SIFHR Checklist
Source: PLoS Med. 2021 Sep 13;18(9):e1003788. doi: 10.1371/journal.pmed.1003788 (PMC8475987; doi:10.1371/journal.pmed.1003788)
Supplement: S4 Text — (DOCX) [file pmed.1003788.s004.docx]

**S4 Text. Feedback obtained from the Delphi surveys**

| **Feedback obtained from the Delphi surveys**  We received feedback from survey participants in three rounds which was used to further refine the research checklist. Below, we present some themes and points received from the three rounds. |
| --- |
| Round oneIn the first round, comments were around providing more details on the methodology, including examples and fine-tuning key definitions. Some examples are listed  - - Further elaborations about the project steps in the preamble and explain the methodology, step by step of the project.  Include 1 or 2 Social innovation examples  - - Identify and describe the end user (i.e., the primary beneficiaries of the social innovation) as well as the problem or challenge."  Include definition of "social innovation provider"  - - Overall cost of the innovation to be included and how finance has been procured  Include limitations added as an additional item? What are the drawbacks? if any,  - - further define a "local stakeholder". |
| Round TwoIn round two, feedback provided we focused on the social innovation impacts, strength and limitations and end user perspectives.  - - Describe the problem identified by end user and addressed by the social innovation from the perspective of the end user  Clarify specific factors that facilitated or limited the implementation of innovation  - - Good to have short term and long-term impacts based on time since the "inputs" were implemented  Focus on the targeted impact of the innovation first and then look at the other unintended impact/effects noted or observed  - - Addition of strengths and future opportunities |
| Round ThreeThe third and final round of the Delphi survey was focused on presentation and format of the research checklist  - The format of how this content is presented needs to be consistent so the user is not distracted  Use vulnerable instead of “marginalized groups” in stakeholder involvement item  - Take out all questions and turning them into statements  Adding a list of past innovations with contact information for all the hubs  - Include examples of Social Innovations from each of the hubs |
