# Supplementary material for: Social Innovation For Health Research: Development of the SIFHR Checklist
Source: PLoS Med. 2021 Sep 13;18(9):e1003788. doi: 10.1371/journal.pmed.1003788 (PMC8475987; doi:10.1371/journal.pmed.1003788)
Supplement: S5 Text — SIFHR, Social Innovation For Health Research. (PDF) [file pmed.1003788.s005.pdf]

ITEM NO.	ITEM	DESCRIPTION	REPORTED ON PAGE NO.
1	Brief Name	The title or abstract identified of this social innovation in health research study.	______
2	Problem	Describe the current context, background and problem addressed by the social innovation from the perspective of the end user.	______
3	Rationale	Describe the rationale for the social innovation, including factors that show a change is needed from the perspective of the end user.	______
4	Social Innovation	Describe the key components of the social innovation. This could be accompanied by a detailed description, a photograph, or a figure. Describe each of the processes, activities, and elements used in the social innovation, including any enabling or supporting activities.	______
5	End Users	Describe the end users of the social innovation in health. Describe how end users are also direct or indirect beneficiaries of the social innovation.	______
6	Stakeholder Involvement	Describe how local stakeholders, including end users, are involved in design, development, implementation, and evaluation of the social innovation in health. In addition, describe the role of marginalized/vulnerable individuals or groups (e.g., people with disability or others as defined by the innovators) in these processes.	______
7	Inputs	Describe any physical, digital or informational materials used or distributed during training, delivery and/or implementation of in the social innovation; provide information on where the materials can be accessed† (e.g. online, appendix, URL).	______
8	Provider	For each category of the social innovation provider (e.g. community member, trained layperson, other individual), describe their expertise, background, role and any specific training given.	______
9	Implementation Strategy	Describe the implementation strategy for the social innovation and whether it is delivered individually, as a group, or partnership. Describe the level of external resources for implementation (e.g., internet access). Describe the frequency and duration of the social innovation delivery.	______

ITEM NO.	ITEM	DESCRIPTION	REPORTED ON PAGE NO.
10	Monitoring & Evaluation Strategy	Describe what is measured, how, and when as part of monitoring and evaluation. This includes measurement of health, social, and other impacts.	_______
11	Setting	Describe the population, type(s) of location(s) where the social innovation is delivered, including any necessary social, political, cultural, environmental or other contextual issues. Describe at what level the innovation is implemented (e.g., district, subdistrict, village). This includes a description of the online setting for online social innovation.	_______
12	Adaptability	Consider how the social innovation could be adapted, scaled up, or used in contexts other than the one described, if appropriate.	_______
13	Financing	Describe how the social innovation in health has been funded at design, development, implementation, and evaluation stages. Describe how the social innovation could generate revenue (if applicable) or be institutionalized (if applicable) in order to be sustained in the future.	_______
14	Health Impact	Describe the health impact of the social innovation over a period of time and the methods to assess health impact. Health is defined broadly here according to the WHO definition.	_______
15	Social Impact	Describe the non-medical impact of the social innovation over a period of time. This could be impact on the environment, social changes, or other non-medical impact (e.g. lessons learned, new processes that emerged from the project, new relationships and networks, application of learned processes to other problems).	_______
16	Limitations	Describe the limitations and potential unintended consequences of the social innovation in health during the design, development, or implementation.	_______
17	Strengths	Describe how the social innovation in health improves on conventional practice.	_______

SOCIAL INNOVATION IN HEALTH INITIATIVE

SESH Crowdsourcing For Health

TDR For research on diseases of poverty
UNICEF • UNDP • World Bank • WHO
